# wEight chanGes, caRdio-mEtabolic risks and morTality in patients with hyperthyroidism (EGRET): a protocol for a CPRD–HES linked cohort study

**DOI:** 10.1136/bmjopen-2021-055219

**Published:** 2021-10-01

**Authors:** Barbara Torlinska, Jonathan M Hazlehurst, Krishnarajah Nirantharakumar, G Neil Thomas, Julia R Priestley, Samuel J Finnikin, Philip Saunders, Keith R Abrams, Kristien Boelaert

**Affiliations:** 1 Institute of Applied Health Research, University of Birmingham, Birmingham, UK; 2 NIHR Birmingham Biomedical Research Centre, University Hospitals Birmingham NHS Foundation Trust, Birmingham, UK; 3 Department of Diabetes and Endocrinology, University Hospitals Birmingham NHS Foundation Trust, Birmingham, UK; 4 Centre for Endocrinology, Diabetes and Metabolism, Birmingham Health Partners. University Hospitals Birmingham NHS Foundation Trust, Birmingham, UK; 5 Midlands Health Data Research UK, University of Birmingham, Birmingham, UK; 6 British Thyroid Foundation, Harrogate, UK; 7 Ridgacre Medical Centres, Birmingham, UK; 8 Department of Statistics, University of Warwick, Coventry, UK; 9 Centre for Health Economics, University of York, York, UK

**Keywords:** thyroid disease, cardiac epidemiology, epidemiology

## Abstract

**Introduction:**

Hyperthyroidism is a common condition affecting up to 3% of the UK population. Treatment improves symptoms and reduces the risk of atrial fibrillation and stroke that contribute to increased mortality. The most common symptom is weight loss, which is reversed during treatment. However, the weight regain may be excessive, contributing to increased risk of obesity. Current treatment options include antithyroid drugs, radioiodine and thyroidectomy. Whether there are differences in either weight change or the long-term cardiometabolic risk between the three treatments is unclear.

**Methods and analysis:**

The study will establish the natural history of weight change in hyperthyroidism, investigate the risk of obesity and risks of cardiometabolic conditions and death relative to the treatment. The data on patients diagnosed with hyperthyroidism between 1 January 1996 and 31 December 2015 will come from Clinical Practice Research Datalink linked to Hospital Episode Statistics and Office of National Statistics Death Registry. The weight changes will be modelled using a flexible joint modelling, accounting for mortality. Obesity prevalence in the general population will be sourced from Health Survey for England and compared with the post-treatment prevalence of obesity in patients with hyperthyroidism. The incidence and time-to-event of major adverse cardiovascular events, other cardiometabolic outcomes and mortality will be compared between the treatments using the inverse propensity weighting model. Incidence rate ratios of outcomes will be modelled with Poisson regression. Time to event will be analysed using Cox proportional hazards model. A competing risks approach will be adopted to estimate comparative incidences to allow for the impact of mortality.

**Ethics and dissemination:**

The study will bring new knowledge on the risk of developing obesity, cardiometabolic morbidity and mortality following treatment for hyperthyroidism to inform clinical practice and public health policies. The results will be disseminated via open-access peer-reviewed publications and directly to the patients and public groups (Independent Scientific Advisory Committee protocol approval #20_000185).

**Strengths and limitations of this study**

- Rich data from a large sample allowing for pre-exposure history and long follow-up, spanning across primary and secondary care.
- Contextualisation of post-treatment obesity prevalence by comparison with the general population adjusted for sex and age.
- A broad range of patient-relevant cardiometabolic outcomes will be evaluated.
- Large real-world observational study of treatment options not amenable to randomised clinical trials.
- The study’s main limitations are related to the intrinsic nature of the real-world data, such as the secondary use of data or the presence of missing data.

## Introduction

Hyperthyroidism is a common condition affecting 3% of women and 0.3% of men in the UK.^1^ Common clinical features indicating hyperthyroidism include weight loss, heat intolerance, tremor, palpitations and anxiety.^2^ Treatment is critical to minimise complications including atrial fibrillation and stroke that contribute to the observed 20% increase in mortality.^3^

There are three treatment options: antithyroid drugs (ATDs), radioactive iodine (I-131, radioiodine) or surgical, either as a total or hemithyroidectomy. ATDs are associated with a high rate of relapse (30%–70%),^4^ while treatment with radioiodine or thyroidectomy leads to the development of hypothyroidism, requiring lifelong levothyroxine replacement, which is seen in 80% of patients administered radioiodine and up to 100% undergoing thyroidectomy. Guidance recommends discussion of all three options with the patient.^5 6^ There is insufficient evidence to recommend one treatment over the others, although some studies indicate differences in cardiac events and mortality between the treatment modalities.^7 8^

Weight gain following various treatments for hyperthyroidism has been described.^9 10^ Clinicians commonly assume that the observed weight gain is a simple regain of weight lost prior to the initiation of treatment. However, an analysis of 1373 patients with hyperthyroidism has demonstrated a higher prevalence of obesity at 3 years of follow-up when compared with the age-matched and sex-matched background population (37% vs 26% in men, p<0.001; 32% vs 26% in women, p<0.001).^11^ These findings suggest excess weight gain beyond patients’ premorbid weight (weight overshoot). Additionally, the study showed significant differences in final weight gain compared with baseline between those treated with ATDs (5.4 kg, 95% CI 4.8 to 6.0) and those who received radioiodine but did not develop hypothyroidism (5.1 kg, 95% CI 4.3 to 6.1) when compared with those treated with radioiodine who developed subsequent hypothyroidism (7.1 kg, 95% CI 6.6 to 7.7). The extent of weight change in patients treated with thyroidectomy was not evaluated.

Prior to diagnosis, the majority of patients with hyperthyroidism notice that they lose weight, often despite increased appetite, due to the regulatory effects of thyroid hormones on metabolism. As thyroid hormone levels normalise following treatment, weight change may be observed. While weight gain following treatment is common, it may become a major psychological stressor and can affect treatment compliance. Untreated hyperthyroidism may result in serious consequences, including thyroid storm, heart failure, embolic events, atrial fibrillation, osteoporosis, muscle weakness, neuropsychiatric symptoms and, rarely, cardiovascular collapse and death.^12^ Patients need to be appropriately counselled about the possibility of significant weight gain associated with the treatment of hyperthyroidism. However, there are currently insufficient data available about weight overshoot, cardiovascular risk and treatment modality selection to provide clear guidance to our patients. In our recent survey of British Thyroid Association members, of 35 clinicians who responded, only 23% usually discuss weight gain with patients treated for hyperthyroidism despite 70% of respondents feeling this was a significant clinical problem.

We recently surveyed doctors and dieticians and found that while there was agreement that weight gain following treatment for hyperthyroidism represents a medical problem, there is no consensus about the significance of or the approach to this anticipated weight gain. When surveyed, dieticians expressed concerns that they are not equipped to advise on dietary issues, given the switch from a highly catabolic state (weight loss state) in untreated hyperthyroidism to a situation of anticipated weight gain following treatment.

As previously shown, hyperthyroidism is associated with increased cardiovascular morbidity and mortality, which are not completely reversed by treatment.^7 8 13 14^ Among cardiovascular comorbidities, atrial fibrillation is the most common, occurring in 2%–20% of untreated patients,^15^ and complications including heart failure and thromboembolic events are observed in patients with hyperthyroidism. In a recent meta-analysis, an increased risk of atrial fibrillation with higher concentrations of thyroid hormones at presentation was confirmed.^16^ In a nationwide register study with a mean follow-up time of 10 years, an increased risk of all arrhythmias was found in patients undergoing treatment with radioiodine or thyroidectomy when compared with controls with no thyroid dysfunction.^8^ Patients with subclinical hyperthyroid and hypothyroid dysfunctions were found to have increased risks of heart failure (HR=1.94, 95%CI 1.01 to 3.72 for thyroid-stimulating hormone (TSH) <0.10 mIU/L and HR=1.86, 95% CI 1.27 to 2.72 for TSH >10.0 mIU/L) in a pooled analysis of individual participant data using available prospective cohorts with thyroid function tests and subsequent follow-up of heart failure.^17^ In a large cohort of UK patients on levothyroxine replacement, mortality was increased in the lowest (<0.1) and highest TSH categories (>4.0) compared with 2.0–2.5 mIU/L, while risks of ischaemic heart disease and heart failure were found to increase at high concentrations of TSH (>10.0 mIU/L).^18^

There is insufficient evidence to systematically review the differences in health outcomes between the three different treatments. Due to limitations arising from the need to adhere to radiation protection and varied requirements of the procedures (a prolonged course of medication, one-off radioiodine tablet or surgical procedure), randomisation and blinding between all three modalities is often not possible, making randomised controlled trials nearly non-existent in this area of research.

At our recent patient engagement event, patients felt that there was insufficient information to help guide the choice of treatment. They were concerned about the risk of excessive weight gain and its long-term cardiometabolic consequences and frustrated with the lack of information, allowing them to prioritise one treatment over the other. There was also disappointment about the lack of support services addressing weight and diet across treatment for hyperthyroidism. Patients were particularly concerned about the perceived risk of weight gain associated with radioactive iodine and agreed that proper quantification of weight gain and cardiovascular risks across the treatments would influence their therapy preferences and could inform anticipatory dietary modification.

### Aims and objectives

The overall aim of this study was to investigate the risks of gaining weight and developing obesity, developing cardiometabolic conditions or death following treatment for hyperthyroidism and to compare these risks between the three treatment modalities.

#### Primary objective

The primary aim of this study was to assess the effect of type of treatment on weight changes during and after therapy for hyperthyroidism. The post-treatment prevalence of obesity will also be evaluated and compared with that of the general population.

#### Secondary objectives

Further, the effect of treatment and weight status on the incidence of major adverse cardiovascular events (MACEs), other cardiometabolic outcomes and mortality following each treatment modality will be evaluated.

## Methods

This is a retrospective, longitudinal, observational study using routinely collected data. Data for the study will come from the Clinical Practice Research Datalink (CPRD), linked to Hospital Episode Statistics (HES) admissions and outpatient databases. The study is to be conducted between 1 February 2021 and 31 July 2022. The protocol was approved by the Independent Scientific Advisory Committee (ISAC #20_000185).

### Data sources

CPRD collects and anonymises patient electronic health record data from a network of general practitioner (GP) practices across the UK.^19^ Although the records in the database cover all four UK countries, HES-linkable records are limited to those from England only. Two HES datasets will be used: HES outpatient and HES admitted patient care. Linkage to HES databases is required to obtain the data, which may be missing from the GP records on non-medical treatment for hyperthyroidism (radioactive iodine and thyroidectomy). Additionally, data on MACEs will also be sourced from HES, as such events are typically being treated in the hospital setting.

Linkage to social data is required to adjust the analysis for socioeconomic status, which is a known risk factor for obesity and increased mortality. This will be obtained as the Index of Multiple Deprivation (IMD). Linkage to Office of National Statistics data is required to get access to the date of death and cause of death. Prevalence of post-treatment obesity will be compared with the background population sourced from Health Survey for England, a yearly survey monitoring trends in the nation’s health and care.

### Population

Adult patients (≥18 years) who were for the first time diagnosed with hyperthyroidism (the list of codes in online supplemental appendix) between 1 January 1996 and 31 December 2015 will be eligible for inclusion. To minimise missed cases, the diagnosis will be additionally identified by a prescription of carbimazole or propylthiouracil, which are ATDs indicated only for treatment of hyperthyroidism. A minimum of 12 months of data must be available before the date of diagnosis and after the index date. CPRD records need to be classified as acceptable research quality.

10.1136/bmjopen-2021-055219.supp1 Supplementary data

Exclusion criteria encompass ATD treatment of duration shorter than 6 months as the only treatment, that is, with no radioactive iodine or thyroidectomy followed. This is to avoid contamination by misdiagnosis or spontaneously resolving thyroiditis. Patients treated with both definitive treatment methods, that is, radioiodine and thyroidectomy, will also be excluded. However, ATD treatment followed by either radioiodine or thyroidectomy (one of the definitive treatments) is considered a pretreatment and does not constitute an exclusion criterion.

### Exposure

The exposure in the study is the treatment for hyperthyroidism. The study patients will be divided into three groups based on the treatment administered: (1) radioiodine: the allocation to this treatment arm will be assigned based on the Read code or OPCS-4 code of the treatment procedure for radioiodine; (2) thyroidectomy: the allocation to this treatment arm will be assigned based on the Read code or OPCS-4 code of the surgical procedure; (3) medical treatment arm will be identified based on the absence of radioiodine treatment code and thyroidectomy code in the presence of ATD treatment (based on the Read code or British National Formulary (BNF) code) longer than 6 months.

We anticipate good quality of ATD prescription and thyroidectomy. However, radioiodine, typically administered in outpatients, may be missed in a substantially high proportion of patients. Since ATD treatment does not incur permanent hypothyroidism, the long-term prescription of levothyroxine for hypothyroidism following treatment with ATD alone, that is, without any definitive procedures, will be considered as a proxy for radioiodine administration.

### Outcomes

The primary outcome is weight change (in kilogram) during and following the treatment for hyperthyroidism, as well as prevalence of obesity following the treatment. The secondary outcomes include cardiometabolic events and mortality as presented in table 1.

**Table 1:** Secondary outcomes and their data sources

Secondary outcome	Source
MACE (cardiovascular death, nonfatal myocardial infarction or non-fatal stroke)	HES
Type 2 diabetes mellitus	CPRD
Congestive heart failure	HES
Ischaemic heart disease	HES
Stroke and transient ischaemic attack	HES
Cardiovascular mortality	ONS
All-cause mortality	ONS

CPRD, Clinical Practice Research Database; HES, Health Episode Statistics; MACE, major adverse cardiovascular event; ONS, Office for National Statistics.

### Confounding

**Figure 1:**
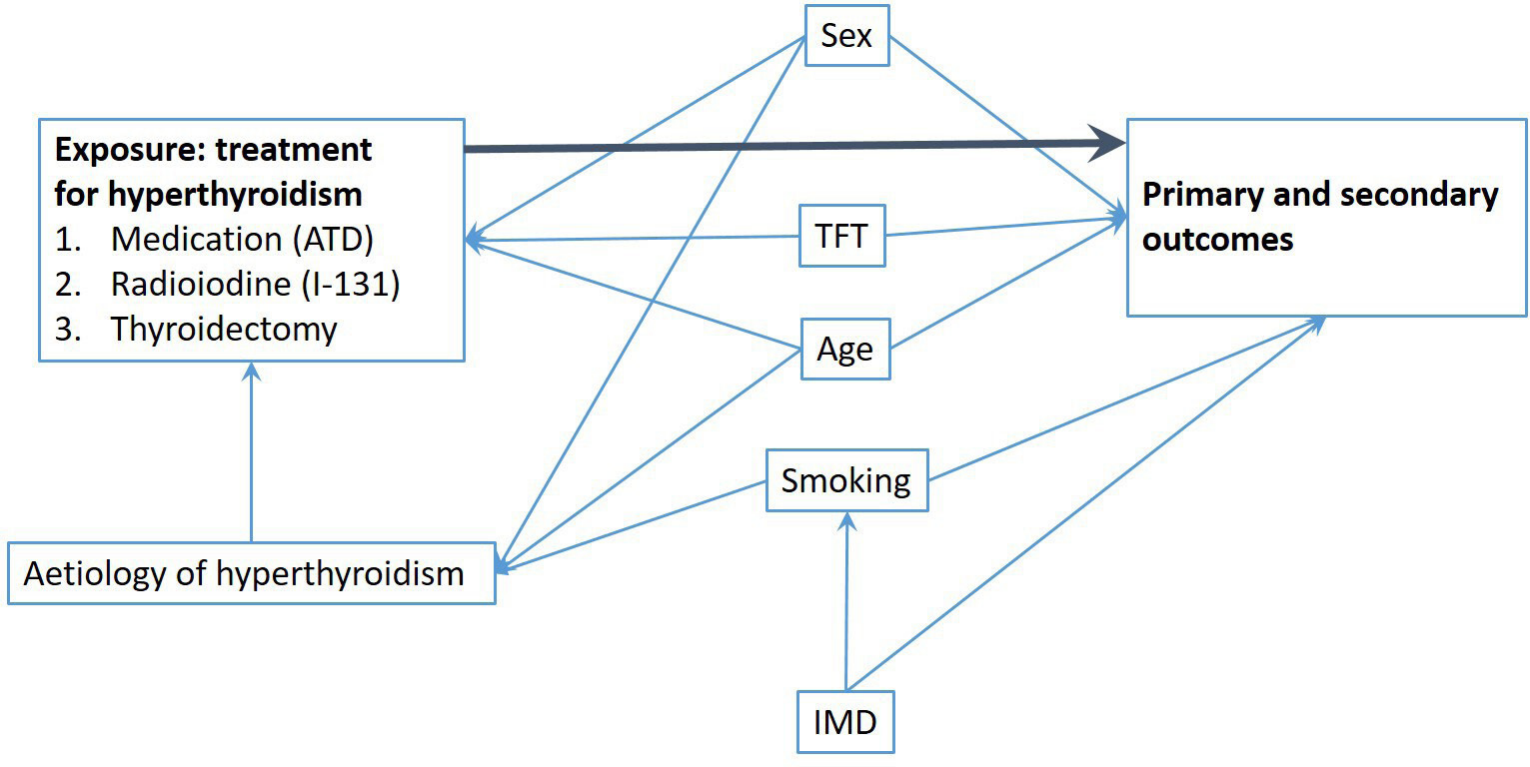
Directed acyclic graph illustrating confounding in the study. The wide dark arrow indicates the relationship of interest. ATD, antithyroid drugs; IMD, Index of Multiple Deprivation; TFT, thyroid function test.

Based on the literature and clinical experience, we identified a number of confounders affecting both the exposure and the outcomes of our study. The modelled outcomes will be adjusted for baseline data (sex, age, IMD quartiles, smoking status and comorbidities) and time-varying covariates (thyroid function, cumulative time on the medical treatment, time since diagnosis, levothyroxine replacement, pregnancy and diagnosis of cancer). Table 2 presents the details of how covariates are defined and handled. Figure 1 depicts the directed acyclic graphs representing the relationship between the covariates. We will explore the relationships between the potential confounders and both exposures and outcomes independently in order to establish their potential role in this particular dataset.

**Table 2:** Definitions of study covariates

Variable	Definition
Aetiology of hyperthyroidism	Identified based on Read codes and categorised in (1) Graves’ disease, (2) toxic nodular goitre and (3) undefined. The undefined category will consist of unspecified diagnosis and missing aetiology data.
Age	Age at index date
Baseline fT4	The highest measurement between 3 months prior to diagnosis and the index date
BMI	Normal (or underweight) <25 kg/m^2^, overweight 25–30 kg/m^2^ and obese ≥30 kg/m^2^; additionally, BMI status will be deduced from the Read code
Cancer diagnosis	Switch-type binary variable assuming lifelong status of cancer comorbidity
Frequency of GP visits	Time-varying variable of number of GP visits in a unit of time
IMD	IMD will be stratified into quintiles.
Levothyroxine (LT4) replacement	Switch-type binary variable assuming lifelong LT4 administration following development of hypothyroidism in radioiodine and thyroidectomy treatment groups
Pregnancy	Time-varying binary variable recording pregnancy
Pretreatment ATD	Cumulative time on ATD between diagnosis and the index date
Sex	Binary variable
Smoking status	As recorded at the index date, last observation carried forward if not available at the index date
TSH	Serial TSH, log-transformed for the analysis
Time since diagnosis	Time-varying variable measuring cumulative time since diagnosis

ATD, antithyroid drug; BMI, body mass index; GP, general practitioner; IMD, Index of Multiple Deprivation; TSH, thyroid-stimulating hormone.

### Index date and follow-up

The index date in the cohort is the date of initiation of treatment for hyperthyroidism, which defines the treatment group (either ATD, radioiodine or thyroidectomy). There is a requirement of at least 12 months’ data available prior to the index date.

The natural history of weight changes will be modelled preindex and postindex date. While the follow-up for the analysis of outcomes will start at the index date, a minimum of 12-month follow-up is required for the inclusion.

### Data analysis

#### Natural history of weight changes

Descriptive statistics will provide summaries about the sample and weight as the outcome measure.

The natural history of weight changes will be modelled longitudinally. The model will estimate the weight (kilogram) over three stages: premorbid, duration of treatment and post-treatment. Premorbid weight is defined as body weight recorded at least 3 months before diagnosis or treatment initiation (whichever comes first). The treatment phase will start from the time of diagnosis or the first prescribed dose or procedure and will last until 3 months after the last recorded date of treatment. Any weight recoded thereafter will be considered as post-treatment.

Only patients having at least one weight record (quantitative or qualitative) in each of the three phases of the condition (premorbid, treatment and post-treatment) will be included in the natural history of weight analysis. The extent of missing data will be reported. As with any real-world data, we anticipate a relatively high proportion of intermittent missing weight data. This missingness can be assumed to be missing at random conditional on other covariates.^20^ Hence, the missing values will be imputed using the multiple imputation by chained equations (MACE).^21^ To improve precision of the imputation, the existing qualitative information from Read codes stating the direction of change (eg, ‘weight increasing (1622.00)’) or body mass index (BMI) status (eg, ‘overweight (22AA.00)’) together with other demographic and clinical covariate data will be entered into the imputation regression model.

Additionally, in order to allow for the fact that patients may die, and their weight trajectories will be subject to informative dropout due to death, we will model weight changes using a flexible joint modelling approach.^22^ Effectively, the analysis of weight history will combine three techniques: (1) complete-case analysis (including only patients with at least one, either quantitative or qualitative, weight status code in each of the phases); (2) imputation of weight where qualitative codes are present; and (3) joint modelling to incorporate the informative missingness due to death. To check the sensitivity of the correctly specified imputation model, a fully Bayesian approach that jointly imputes missing values and estimates the parameters of the longitudinal model will also be conducted.^23–25^ Weight in all three treatment groups will be modelled simultaneously; the ATD group will be used as the reference group. Any marginal pairwise comparisons will be corrected for multiple testing by applying Bonferroni correction.

Further, the sensitivity to death and to missed treatment coding will be checked. During our meetings with the patients, we were informed that weight changes are important but are not of the utmost priority. In the face of death or any serious illness, weight maintenance loses its importance. Hence, our interest in weight changes in an immortal cohort, and thus, we would like to model weight in patients in such health that allows them to survive to the end of the study. Patients who died during the study will be excluded.

#### Prevalence of obesity

Post-treatment obesity in patients will be defined as ≥30 kg/m^2^, in line with the WHO definition,^26^ or identified from the Read codes when quantitative values are not available. The prevalence of obesity will be assessed in the entire cohort and stratified by the treatment group.

The post-treatment BMI will be compared with those in the background population (Health Survey for England). Two measures of effect will be investigated: (1) difference in overall BMI (kg/m^2^) and (2) difference in proportions of obesity. The effects will be tested with linear regression and logistic regression, respectively, in reference to the background population. The analyses will be adjusted for age and stratified by sex. Only complete case analysis will be conducted.

#### Cardiometabolic risks and mortality

The descriptive analysis of primary and secondary outcomes will provide useful summaries about the sample and the outcome measures. Besides the unadjusted descriptive statistics, such as the incidence rate of each outcome, simple graphics analysis will be provided.

Causal inference in the analysis of cardiometabolic outcomes and mortality will be undertaken using inverse propensity weighting (IPW). This method was empirically shown to be superior to other propensity score-based approaches in analysis of multiple treatments with a binary outcome.^27^ The propensity-score matching, even though well valuated in two-arm treatment, in multiple treatments might introduce bias; the IPW is more efficient in such scenario. Additionally, IPW method is more flexible and requires weaker unconfoundedness assumption.^28^

Generalised propensity weights with treatment category as dependent variable will be calculated in the multinominal logistic model.^29^ The model will be developed using the following covariates: age at the index date, sex, baseline serum fT4, aetiology of hyperthyroidism, smoking status, IMD, cumulative time on ATD between diagnosis and the index date, time since diagnosis and BMI status.

Variables related to treatment, as an outcome, at p<0.10 will be selected for inclusion in the multiple propensity weights.^30^ To identify these variables, we will conduct several regression analyses with the treatment as a dependent and each potential confounder as an independent variable. Furthermore, it will be investigated whether adding interaction terms or higher-order terms for continuous variables will improve the balance of the model. The Hausman test will be used to check the independence of irrelevant alternatives assumption, which is the main assumption of multinomial regression analysis.^30^ If this assumption is not met, multinomial probit analysis will be used instead.

The estimation of propensity weights will be conducted by the method proposed by McCaffrey *et al*.^31 32^ The appropriateness of IPW analysis will be assessed by checking the overlap (positivity assumption). According to the positivity assumption, each patient should have a non-zero probability of being indicated to each treatment category. Lack of overlap in the distribution of observed pretreatment characteristics between groups receiving different treatment indicates the positivity assumption is violated. This overlap will be checked visually. If poor overlap is identified, we will use Rubin’s trimming method,^33^ allowing discarding non-overlapping cases.

Further, pairwise balance in the distribution of all included variables between the three treatment groups will be tested with linear regression for continuous variables, logistic regression for binary and multinomial logistic regression for nominal variables. The analysis will be done without and with adjustments. The propensity weights will be considered balanced if there are no statistically significant (p<0.05) differences between the likelihood of receiving a different type of treatment. If imbalances remain after weighting, a doubly robust estimation approach will be applied^32^; that is, the imbalanced variables will be added to the model.

While IPW will adjust for confounding at baseline, other time-varying adjustments will be applied to correct for the events between the baseline and the outcome. The proposed covariates are treatment with levothyroxine for developed hypothyroidism, log TSH levels and informative observations of number of GP visits in a unit of time.

Incidence rate ratios of outcomes will be calculated with Poisson regression and modelled adjusting for time-varying covariates as listed further. Time-to-event analysis with adjustments will be modelled. The appropriate model will be applied, depending on the proportional hazard assumption’s results: if the assumption holds—Cox proportional hazard regression will be used; if it is violated, alternative techniques such as time-varying measures in extended Cox models will be applied.

Proposed time-varying adjustments for both analyses will include postindex date data, that is, treatment with levothyroxine for developed hypothyroidism, logTSH levels and informative observations of number of GP visits in a unit of time. To allow for the fact that patients may die, a competing risks approach will be adopted for estimating comparative incidences for non-fatal events to allow for the impact of mortality as a competing risk.^22^

#### Sensitivity to missed radioiodine record

Whenever ATD patients develop permanent hypothyroidism, a radioiodine treatment will be assumed, as explained in the Exposure section. All analyses will be repeated to check sensitivity to this assumption, excluding patients in the ATD group who developed hypothyroidism following medical treatment being the only antithyroid treatment on record.

### Patient and public involvement

Our patients with hyperthyroidism have been pivotal in formulating the research questions and informing the study design. The study idea originated from listening to patients and their concerns. As part of the consultation process, we held a meeting, advertised locally and nationally via the British Thyroid Foundation (BTF), with patients who had either previously been treated or were currently being treated for hyperthyroidism. During an open discussion, key themes were identified as important to patients across the treatment course of hyperthyroidism. At diagnosis, patients often reported that there was insufficient information to determine which treatment choice would be best for them, particularly regarding long-term treatment consequences; hence, a major driver of the study design is the examination of long-term cardiometabolic outcomes. The comment that “radioactive iodine will make me fat” was repeatedly mentioned in the group discussion. We were already interested in the potential issue of weight gain beyond premorbid weight, and this is something we will be investigating in this project. The main themes reported were that in addition to insufficient information to make a clear, informed treatment choice, patients did not feel that they were suitably counselled about weight regain nor that there was a mechanism in place to predict or modulate this potential effect. The insufficiency of information on the treatment choices and their long-term consequences was further confirmed in a BTF survey involving 353 patients. Among responders who received definitive treatment (n=167) who therefore had knowledge of postdefinitive treatment procedure effects, a third would have not decided to proceed with their choice of treatment.

To continue guidance by patients, we have formed a patient group, which was engaged at the design stage and will continue to advise us during the project. Additionally, a BTF representative (JP) joined our team as a coapplicant to represent patients’ voices regularly throughout the research process. As the study progresses, the patient group and the patients’ representative will be involved in rationalising the design if any challenges are encountered, in discussing the implications and relevance of the emerging results, and in finalising the key messages of the study to facilitate patient-centred dissemination. We will involve our patient partners in the dissemination of the results and the development of future avenues of research.

Additional guidance comes from the GP advisory group (SJF and PS), who has shared its experience and expertise from the front-end of data collection. This was especially important while establishing the feasibility of modelling and interpreting the weight changes based on the already recorded weight entries. The GP advice will be further used in the interpretation of results, dissemination process and the planning of further initiatives.

## Ethics and dissemination

The study protocol has been approved by the ISAC in March 2021 (#20_000185). ISAC is a non-statutory expert advisory body established in 2006 by the Secretary of State for Health to provide scientific advice on research requests to access data provided by CPRD.

The primary aim was to inform clinical practice within the NHS and to provide evidence for future research in managing long-term consequences of hyperthyroidism and its treatment. The dissemination plan targets audiences at various levels: clinical and academic as well as patients and public. To address the needs of the former, we are planning to share the findings at the scientific endocrinology meetings and to publish them in leading open-access peer-reviewed academic journals. The BTF, a national community of patients with thyroid conditions in the UK, will play a key role in disseminating the results of the study to the public, patients and their families and carers. The BTF will share results via traditional and social media channels.

## Supplementary Material

Reviewer comments

Author's manuscript

## Data Availability

No data are available.
