# Supplementary material for: wEight chanGes, caRdio-mEtabolic risks and morTality in patients with hyperthyroidism (EGRET): a protocol for a CPRD–HES linked cohort study
Source: BMJ Open. 2021 Oct 1;11(10):e055219. doi: 10.1136/bmjopen-2021-055219 (PMC8488707; doi:10.1136/bmjopen-2021-055219)
Supplement: Supplementary data [file bmjopen-2021-055219supp001.pdf]

**APPENDIX: Medical codes used for data search**

**Table 1:** Read codes for diagnosis of hyperthyroidism

| Read code | Description |
|---|---|
| C02..00 | Thyrotoxicosis |
| C02..11 | Hyperthyroidism |
| C02..12 | Toxic goitre |
| C020.00 | Toxic diffuse goitre |
| C020.11 | Basedow's disease |
| C020.12 | Graves' disease |
| C020000 | Toxic diffuse goitre with no crisis |
| C020100 | Toxic diffuse goitre with crisis |
| C020200 | Thyroid-associated dermopathy |
| C020z00 | Toxic diffuse goitre NOS |
| C021.00 | Toxic uninodular goitre |
| C021000 | Toxic uninodular goitre with no crisis |
| C021100 | Toxic uninodular goitre with crisis |
| C021z00 | Toxic uninodular goitre NOS |
| C022.00 | Toxic multinodular goitre |
| C022000 | Toxic multinodular goitre with no crisis |
| C022100 | Toxic multinodular goitre with crisis |
| C022z00 | Toxic multinodular goitre NOS |
| C023.00 | Toxic nodular goitre unspecified |
| C023000 | Toxic nodular goitre unspecified with no crisis |
| C023100 | Toxic nodular goitre unspecified with crisis |
| C023z00 | Toxic nodular goitre NOS |
| C024.00 | Thyrotoxicosis from ectopic thyroid nodule |
| C024000 | Thyrotoxicosis from ectopic thyroid nodule with no crisis |
| C024100 | Thyrotoxicosis from ectopic thyroid nodule with crisis |
| C024z00 | Thyrotoxicosis from ectopic thyroid nodule NOS |
| C02y.00 | Thyrotoxicosis of other specified origin |
| C02y.11 | Factitia thyrotoxicosis |
| C02y000 | Thyrotoxicosis of other specified origin with no crisis |
| C02y100 | Thyrotoxicosis of other specified origin with crisis |
| C02y200 | Thyrotoxicosis factitia |
| C02y300 | Thyroid crisis |
| C02yz00 | Thyrotoxicosis of other specified origin NOS |
| C02z.00 | Thyrotoxicosis without mention of goitre or other cause |
| C02z000 | Thyrotoxicosis without mention of goitre or cause no crisis |
| C02z100 | Thyrotoxicosis without mention of goitre, cause with crisis |
| C02zz00 | Thyrotoxicosis NOS |

**Table 2:** Codes for the treatment group allocation

| **Treatment group allocation** | **Coding system** | | |
|---|---|---|---|
| | **Read** | **BNF** | **OPCS-4** |
| **Medication:** | | | |
| Carbimazole | | 06.02.02.00 | |
| Propylthiouracil | | 06.02.02.00 | |
| **Radioiodine:** | | | |
| Radioactive drug therapy | 5A16.00 | | X65.5 |
| I131 radiotherapy | 5A16.11 | | |
| Iodine 131 radiotherapy | 5A16.12 | | |
| **Thyroidectomy:** | | | |
| Thyroid gland operations | 711..12 | | B12.8 |
| Thyroidectomy operations | 7110.00 | | |
| Excision of thyroid gland operations | 7110.11 | | |
| Total thyroidectomy | 7110000 | | B08.1 |
| Subtotal thyroidectomy | 7110100 | | B08.2 |
| Bilateral subtotal thyroidectomy | 7110111 | | B08.2 |
| Hemithyroidectomy | 7110200 | | B08.3 |
| Lobectomy of thyroid gland NEC | 7110300 | | B08.4 |
| Isthmectomy of thyroid gland | 7110400 | | B08.5 |
| Partial thyroidectomy NEC | 7110500 | | B08.6 |
| Thyroidectomy NEC | 7110600 | | B08.6 |
| Other specified thyroidectomy | 7110y00 | | B08.8 |
| Thyroidectomy NOS | 7110z00 | | B08.9 |

**Table 3**: Outcomes codes

| Outcome | Coding system | |
|---|---|---|
| | **ICD-10** | **Read codes** |
| obesity | | 22K5.00, 66C..00, 66CZ.00, C38..00, C380.00, C380.00, C380000, C380100, C380200, C380300, C380400, C380500, C38y011, C38z.00, C38z000 |
| nonfatal myocardial infarction (composite of MACE) | I21-I22 | n/a |
| nonfatal stroke (composite of MACE) | I64 | n/a |
| congestive heart failure | I50 | n/a |
| ischaemic heart disease | I20-I25 | n/a |
| stroke and transient ischaemic attack (TIA) | I60-I69 | n/a |
| type 2 diabetes mellitus | E11 | C109* |

MACE: major adverse cardiovascular event; TIA: transient ischemic attack
