# Supplementary material for: wEight chanGes, caRdio-mEtabolic risks and morTality in patients with hyperthyroidism (EGRET): a protocol for a CPRD–HES linked cohort study
Source: BMJ Open. 2021 Oct 1;11(10):e055219. doi: 10.1136/bmjopen-2021-055219 (PMC8488707; doi:10.1136/bmjopen-2021-055219)
Supplement: Author's manuscript [file bmjopen-2021-055219.draft_revisions.pdf]

BMJ Open

BMJ Open is committed to open peer review. As part of this commitment we make the peer review history of every article we publish publicly available.

When an article is published we post the peer reviewers' comments and the authors' responses online. We also post the versions of the paper that were used during peer review. These are the versions that the peer review comments apply to.

The versions of the paper that follow are the versions that were submitted during the peer review process. They are not the versions of record or the final published versions. They should not be cited or distributed as the published version of this manuscript.

BMJ Open is an open access journal and the full, final, typeset and author-corrected version of record of the manuscript is available on our site with no access controls, subscription charges or pay-per-view fees (http://bmjopen.bmj.com).

If you have any questions on BMJ Open's open peer review process please email info.bmjopen@bmj.com

BMJ Open

wEight chanGes, caRdio-mEtabolic risks and morTality in patients with hyperthyroidism (EGRET): a protocol for a CPRD–HES linked cohort study

Journal:	*BMJ Open*
Manuscript ID	bmjopen-2021-055219
Article Type:	Protocol
Date Submitted by the Author:	07-Jul-2021
Complete List of Authors:	Torlinska, Barbara; University of Birmingham, Institute of Applied Health Research<br>Hazlehurst, Jonathan; University of Birmingham, Institute of Applied Health Research; University Hospitals Birmingham NHS Foundation Trust, Department of Diabetes and Endocrinology<br>Nirantharakumar, Krishnarajah; University of Birmingham, Institute of Applied Health Research; University of Birmingham, Midlands Health Data Research UK<br>Thomas, Neil; University of Birmingham, Institute of Applied Health Research<br>Priestley, Julia; British Thyroid Foundation<br>Finnikin, Samuel; University of Birmingham, Institute of Applied Health Research<br>Saunders, Philip; Ridgacre Medical Centres<br>Abrams, Keith; University of Warwick, Department of Statistics; University of York, Centre for Health Economics<br>Boelaert, K; University of Birmingham, Institute of Applied Health Research; University Hospitals Birmingham NHS Foundation Trust, Birmingham Health Partners
Keywords:	Thyroid disease < DIABETES & ENDOCRINOLOGY, Cardiac Epidemiology < CARDIOLOGY, EPIDEMIOLOGY

SCHOLARONE™
Manuscripts

I, the Submitting Author has the right to grant and does grant on behalf of all authors of the Work (as defined in the below author licence), an exclusive licence and/or a non-exclusive licence for contributions from authors who are: i) UK Crown employees; ii) where BMJ has agreed a CC-BY licence shall apply, and/or iii) in accordance with the terms applicable for US Federal Government officers or employees acting as part of their official duties; on a worldwide, perpetual, irrevocable, royalty-free basis to BMJ Publishing Group Ltd ("BMJ") its licensees and where the relevant Journal is co-owned by BMJ to the co-owners of the Journal, to publish the Work in this journal and any other BMJ products and to exploit all rights, as set out in our licence.

The Submitting Author accepts and understands that any supply made under these terms is made by BMJ to the Submitting Author unless you are acting as an employee on behalf of your employer or a postgraduate student of an affiliated institution which is paying any applicable article publishing charge ("APC") for Open Access articles. Where the Submitting Author wishes to make the Work available on an Open Access basis (and intends to pay the relevant APC), the terms of reuse of such Open Access shall be governed by a Creative Commons licence – details of these licences and which Creative Commons licence will apply to this Work are set out in our licence referred to above.

Other than as permitted in any relevant BMJ Author's Self Archiving Policies, I confirm this Work has not been accepted for publication elsewhere, is not being considered for publication elsewhere and does not duplicate material already published. I confirm all authors consent to publication of this Work and authorise the granting of this licence.

For peer review only

wEight chanGes, caRdio-mEtabolic risks and morTality in patients with hyperthyroidism (EGRET): a protocol for a CPRD–HES linked cohort study

Barbara Torlinska,[1,2] Jonathan M. Hazlehurst,[1,3,4] Krishnarajah Nirantharakumar,[1,5] G. Neil Thomas,[1] Julia Priestley,[6] Sam Finnikin,[1] Phil Saunders,[7] Keith R Abrams*,[8,9] Kristien Boelaert*[1,10]

* joint senior authors

[1] Institute of Applied Health Research, University of Birmingham, Birmingham, UK
[2] NIHR Birmingham Biomedical Research Centre, University Hospitals Birmingham NHS Foundation Trust and University of Birmingham, Birmingham, UK
[3] Centre for Endocrinology, Diabetes and Metabolism, Birmingham Health Partners, Birmingham, UK
[4] Department of Diabetes and Endocrinology, University Hospitals Birmingham NHS Foundation Trust, Birmingham, UK
[5] Midlands Health Data Research UK, University of Birmingham, Birmingham, UK
[6] British Thyroid Foundation, Harrogate, UK
[7] Ridgacre Medical Centres, Birmingham, UK
[8] Department of Statistics, University of Warwick, Coventry, UK
[9] Centre for Health Economics, University of York, York, UK
[10] Birmingham Health Partners, Birmingham, UK

Corresponding author:

Dr Barbara Torlinska
B.Torlinska@bham.ac.uk

Keywords: Thyrotoxicosis, Body weight, Obesity, Treatment for hyperthyroidism, Cardiometabolic outcomes, Mortality

For peer review only - http://bmjopen.bmj.com/site/about/guidelines.xhtml

ABSTRACT

Introduction: Hyperthyroidism is a common condition affecting up to 3% of the UK population. Treatment improves symptoms and reduces the risk of atrial fibrillation and stroke that contribute to increased mortality. The most common symptom is weight loss, which is reversed during treatment. However, the weight regain may be excessive, contributing to increased risk of obesity.

Current treatment options include antithyroid drugs, radioiodine and thyroidectomy. Whether there are differences in either weight change or the long-term cardio-metabolic risk between the three treatments is unclear.

Methods and analysis: The study will establish the natural history of weight change in hyperthyroidism, investigate the risk of obesity and risks of cardio-metabolic conditions and death relative to the treatment. The data on patients diagnosed with hyperthyroidism between 01/01/1996 and 31/12/2015 will come from Clinical Practice Research Datalink (CPRD) linked to Hospital Episode Statistics (HES) and ONS Death Registry.

The weight changes will be modelled using a flexible joint modelling, accounting for mortality. Obesity prevalence in the general population will be sourced from Health Survey for England and compared with the post-treatment prevalence of obesity in patients with hyperthyroidism. The incidence and time-to-event of major adverse cardiovascular events (MACE), other cardio-metabolic outcomes and mortality will be compared between the treatments using the inverse propensity weighting model. Incidence rate ratios of outcomes will be modelled with Poisson regression. Time-to-event will be analysed using Cox proportional hazards model. A competing risks approach will be adopted to estimate comparative incidences to allow for the impact of mortality.

Ethics and dissemination: The study will bring new knowledge on the risk of developing obesity, cardio-metabolic morbidity and mortality following treatment for hyperthyroidism to inform the clinical practice and public health policies. The results will be disseminated via open-access peer-reviewed publications and directly to the patients and public groups. (ISAC protocol approval #20_000185)

STRENGTHS AND LIMITATIONS OF THIS STUDY:

- Rich data from a large sample allowing for pre-exposure history and long follow-up, spanning across primary and secondary care
- Contextualisation of post-treatment obesity prevalence by comparison with general population adjusted for sex and age
- A broad range of patient relevant cardio-metabolic outcomes will be evaluated
- Large real world observational study of treatment options not amenable to randomised clinical trials
- The main study's limitations are related to the intrinsic nature of the real-world data, such as the secondary use of data or the presence of missing data

INTRODUCTION

Hyperthyroidism is a common condition affecting 3% of women and 0.3% of men in the UK.[1] Common clinical features indicating hyperthyroidism include weight loss, heat intolerance, tremor,

For peer review only - http://bmjopen.bmj.com/site/about/guidelines.xhtml

For peer review only

palpitations and anxiety.[2] Treatment is critical to minimise complications including atrial fibrillation and stroke that contribute to the observed 20% increase in mortality.[3]

There are three treatment options: antithyroid drugs (ATDs), radioactive iodine (I-131, radioiodine), or surgical either as a total or hemi-thyroidectomy. ATDs are associated with a high rate of relapse (30-70%);[4] while treatment with radioiodine or thyroidectomy leads to the development of hypothyroidism, requiring life-long levothyroxine replacement, which is seen in 80% of patients administered radioiodine and up to 100% undergoing thyroidectomy. Guidance recommends discussion of all three options with the patient.[5,6] There is insufficient evidence to recommend one treatment over the others, although some studies indicate differences in cardiac events and mortality between the treatment modalities.[7,8]

Weight gain following various treatments for hyperthyroidism has been described.[9,10] Clinicians commonly assume that the observed weight gain is a simple regain of weight lost prior to the initiation of treatment. However, an analysis of 1,373 patients with hyperthyroidism has demonstrated a higher prevalence of obesity at three years of follow-up when compared with the age and sex matched background population (37% vs 26% in men, $p<0.001$; 32% vs 26% in women, $p<0.001$).[11] These findings suggest excess weight gain beyond patients' premorbid weight (weight overshoot). Additionally, the study showed significant differences in final weight gain compared to baseline between those treated with ATDs (5.4 kg [95%CI: 4.8–6.0) and those who received radioiodine but did not develop hypothyroidism (5.1 kg [4.3–6.1]) when compared to those treated with radioiodine who developed subsequent hypothyroidism (7.1 kg [6.6–7.7]). The extent of weight change in patients treated with thyroidectomy was not evaluated.

Prior to diagnosis, the majority of patients with hyperthyroidism notice that they lose weight, often despite increased appetite, due to the regulatory effects of thyroid hormones on metabolism. As thyroid hormone levels normalise following treatment, weight change may be observed. While weight gain following treatment is common, it may become a major psychological stressor and can affect treatment compliance. Untreated hyperthyroidism may result in serious consequences, including thyroid storm, heart failure, embolic events, atrial fibrillation, osteoporosis, muscle weakness, neuropsychiatric symptoms, and rarely cardiovascular collapse and death.[12] Patients need to be appropriately counselled about the possibility of significant weight gain associated with the treatment of hyperthyroidism. However, there is currently insufficient data available about weight overshoot, cardiovascular risk and treatment modality selection to provide clear guidance to our patients. In our recent survey of British Thyroid Association members, of 35 clinicians that responded, only 23% usually discuss weight gain with patients treated for hyperthyroidism despite 70% of respondents feeling this was a significant clinical problem.

We recently surveyed doctors and dieticians and found that whilst there was agreement that weight gain following treatment for hyperthyroidism represents a medical problem, there is no consensus about the significance of or the approach to this anticipated weight gain. When surveyed, dieticians expressed concerns that they are not equipped to advise on dietary issues given the switch from a highly catabolic state (weight loss state) in untreated hyperthyroidism to a situation of anticipated weight gain following treatment.

As previously shown, hyperthyroidism is associated with increased cardiovascular morbidity and mortality, which is not completely reversed by treatment.[7,8,13,14] Among cardiovascular comorbidities, atrial fibrillation is the most common, occurring in 2 to 20% of untreated patients[15] and complications including heart failure and thrombo-embolic events are observed in patients with hyperthyroidism. In a recent meta-analysis, an increased risk of atrial fibrillation with higher

For peer review only - http://bmjopen.bmj.com/site/about/guidelines.xhtml

concentrations of thyroid hormones at presentation was confirmed.[16] In a nation-wide register study with a mean follow-up time of 10 years, an increased risk of all arrhythmias was found in patients undergoing treatment with radioiodine or thyroidectomy when compared with controls with no thyroid dysfunction.[8] Patients with subclinical hyper- and hypothyroid dysfunction were found to have increased risks of heart failure (HR=1.94 [95%CI 1.01– 3.72] for TSH <0.10 mIU/L and 1.86 [1.27–2.72] for TSH >10.0 mIU/L) in a pooled analysis of individual participant data using available prospective cohorts with thyroid function tests and subsequent follow-up of heart failure.[17] In a large cohort of UK patients on levothyroxine replacement, mortality was increased in the lowest (<0.1) and highest TSH categories (>4.0) compared with 2-2.5 mIU/L, while risks of ischaemic heart disease and heart failure were found to increase at high concentrations of TSH (>10.0 mIU/L).[18]

There is insufficient evidence to systematically review the differences in health outcomes between the three different treatments. Due to limitations arising from the need to adhere to radiation protection and varied requirements of the procedures (a prolonged course of medication, one-off radioiodine tablet or surgical procedure), randomisation and blinding between all three modalities is often not possible, making randomised controlled trials nearly non-existent in this area of research.

At our recent patient engagement event, patients felt that there was insufficient information to help guide the choice of treatment. They were concerned about the risk of excessive weight gain and its long-term cardio-metabolic consequences and frustrated with the lack of information, allowing them to prioritise one treatment over the other. There was also disappointment about the lack of support services addressing weight and diet across treatment for hyperthyroidism. Patients were particularly concerned about the perceived risk of weight gain associated with radioactive iodine and agreed that proper quantification of weight gain and cardiovascular risks across the treatments would influence their therapy preferences and could inform anticipatory dietary modification.

Aims and objectives:

The overall aim of the study is to investigate the risks of gaining weight and developing obesity, developing cardio-metabolic conditions or death following treatment for hyperthyroidism and to compare these risks between the three treatment modalities.

Primary objective

The primary aim of this study is to assess the effect of type of treatment on weight changes during and after therapy for hyperthyroidism. The post-treatment prevalence of obesity will also be evaluated and compared with that of the general population.

Secondary objectives

Further, the effect of treatment and weight status on the incidence of major cardiovascular events (MACE), other cardio-metabolic outcomes and mortality following each treatment modality will be evaluated.

For peer review only - http://bmjopen.bmj.com/site/about/guidelines.xhtml

METHODS

Data sources

This is a retrospective, longitudinal, observational study using routinely collected data. Data for the study will come from the Clinical Practice Research Datalink (CPRD), linked to Hospital Episode Statistics (HES) admissions and outpatients databases. CPRD collects and anonymises patient electronic health record data from a network of GP practices across the UK.[19] Although the records in the database cover all four UK countries, HES-linkable records are limited to those from England only.

Two HES datasets will be used: HES outpatient and HES admitted patient care. Linkage to HES databases is required to obtain the data, which may be missing from the GP records on non-medical treatment for hyperthyroidism (radioactive iodine and thyroidectomy). Additionally, data on major cardiovascular events (MACE) will also be sourced from HES, as such events are typically being treated in the hospital setting.

Linkage to social data is required to adjust the analysis for socioeconomic status, which is a known risk factor for obesity and increased mortality. This will be obtained as the Index of Multiple Deprivation (IMD). Linkage to Office of National Statistics (ONS) data is required to get access to the date of death and cause of death. Prevalence of post-treatment obesity will be compared with the background population sourced from Health Survey for England, a yearly survey monitoring trends in the nation's health and care.

Population

Adult patients (≥18 years) who were for the first time diagnosed with hyperthyroidism (the list of codes in Appendix) between 01/01/1996 and 31/12/2015 will be eligible for inclusion. To minimise missed cases, the diagnosis will be additionally identified by a prescription of carbimazole or polythiurauracil, which are antithyroid drugs indicated only for treatment of hyperthyroidism. A minimum of 12 months of data must be available before the date of diagnosis and after the index date. CPRD records need to be classified as acceptable research quality.

Exclusion criteria encompass antithyroid drug treatment of duration shorter than six months as the only treatment, i.e. with no radioactive iodine or thyroidectomy followed. This is to avoid contamination by misdiagnosis or spontaneously resolving thyroiditis. Patients treated with both definitive treatment methods, i.e. radioiodine and thyroidectomy, will also be excluded. However, ATD treatment followed by either radioiodine or thyroidectomy (one of the definitive treatments) is considered a pre-treatment and does not constitute an exclusion criterion.

Exposure

The exposure in the study is the treatment for hyperthyroidism The study patients will be divided into three groups based on the treatment administered: (i) Radioiodine: The allocation to this treatment arm will be assigned based on the Read code or OPCS-4 code of the treatment procedure for radioiodine; (ii) Thyroidectomy: The allocation to this treatment arm will be assigned based on the Read code or OPCS-4 code of the surgical procedure; (iii) Medical treatment arm will be

For peer review only - http://bmjopen.bmj.com/site/about/guidelines.xhtml

identified based on the absence of radioiodine treatment code and thyroidectomy code in the presence of ATD treatment (based on the Read code or BNF code) longer than 6 months.

We anticipate good quality of ATD prescription and thyroidectomy. However, radioiodine, typically administered in outpatients, may be missed in a substantially high proportion of patients. Since ATD treatment does not incur permanent hypothyroidism, the long-term prescription of levothyroxine for hypothyroidism following treatment with ATD alone, i.e. without any definitive procedures, will be considered as a proxy for radioiodine administration.

Outcomes

The primary outcome is weight change (in kg) during and following the treatment for hyperthyroidism, as well as prevalence of obesity following the treatment. The secondary outcomes include cardio-metabolic events and mortality as presented in table 1.

Table 1: Secondary outcomes and their data sources.

Secondary outcome	Source
MACE (cardiovascular death, nonfatal myocardial infarction, or nonfatal stroke)	HES
type 2 diabetes mellitus	CPRD
congestive heart failure	HES
ischaemic heart disease	HES
stroke and transient ischaemic attack	HES
cardiovascular mortality	ONS
all-cause mortality	ONS

MACE: major adverse cardiovascular events, HES: Health Episode Statistics, CPRD: Clinical Practice Research Database, ONS: Office for National Statistics

Confounding

Based on the literature and clinical experience, we identified a number of confounders affecting both the exposure and the outcomes of our study. The modelled outcomes will be adjusted for baseline data (sex, age, IMD quartiles, smoking status, comorbidities) and time-varying covariates (thyroid function, cumulative time on the medical treatment, time since diagnosis, levothyroxine replacement, pregnancy and diagnosis of cancer). Table 2 presents the details of how covariates are defined and handled. Figure 1 depicts the directed acyclic graphs representing the relationship between the covariates. We will explore the relationships between the potential confounders and both exposures and outcomes independently in order to establish their potential role in this particular dataset.

Table 2: Definitions of study covariates

Variable	Definition
Aetiology of hyperthyroidism	Identified based on Read codes and categorised in (i) Graves' disease, (ii) toxic nodular goitre, and (iii) undefined. The undefined category will consist of unspecified diagnosis and missing aetiology data.
Age	Age at index date
Baseline fT4	The highest measurement between 3 months prior to diagnosis and the index date

For peer review only - http://bmjopen.bmj.com/site/about/guidelines.xhtml

Body Mass Index (BMI)	Normal (or underweight) <25 kg/m^2, overweight 25-30 kg/m^2, and obese ≥ 30 kg/m^2; additionally BMI status will be deduced from the Read code
Cancer diagnosis	Switch-type binary variable assuming life-long status of cancer comorbidity
Frequency of GP visits	Time-varying variable of number of GP visits in a unit of time
Index of multiple deprivation (IMD)	IMD will be stratified into quintiles
Levothyroxine (LT4) replacement	Switch-type binary variable assuming life-long LT4 administration following development of hypothyroidism in Radioiodine and Thyroidectomy treatment groups
Pregnancy	Time-varying binary variable recording pregnancy
Pre-treatment ATD	Cumulative time on ATD between diagnosis and the index date
Sex	Binary variable
Smoking status	As recorded at the index date; last observation carried forward (LOCF) if not available at the index date
Thyroid Stimulating Hormone (TSH)	Serial TSH, log-transformed for the analysis
Time since diagnosis	Time-varying variable measuring cumulative time since diagnosis

Index date and follow-up

The index date in the cohort is the date of initiation of treatment for hyperthyroidism, which defines the treatment group (either ATD, radioiodine or thyroidectomy). There is a requirement of at least 12 months data available prior to the index date.
The natural history of weight changes will be modelled pre-and post-index date. While the follow-up for the analysis of outcomes will start at the index date; a minimum of 12 month follow-up is required for the inclusion.

DATA ANALYSIS

Natural history of weight changes

Descriptive statistics will provide summaries about the sample and weight as the outcome measure. The natural history of weight changes will be modelled longitudinally. The model will estimate the weight (kg) over three stages: premorbid, duration of treatment, and post-treatment. Premorbid weight is defined as body weight recorded at least 3 months before diagnosis or treatment initiation (whichever comes first). The treatment phase will start from the time of diagnosis or the first prescribed dose or procedure and will last until three months after the last recorded date of treatment. Any weight recoded thereafter will be considered as post-treatment.

Only patients having at least one weight record (quantitative or qualitative) in each of the three phases of the condition (premorbid, treatment, post-treatment) will be included in the natural history of weight analysis. The extent of missing data will be reported. As with any real-world data, we anticipate a relatively high proportion of intermittent missing weight data. This missingness can

For peer review only - http://bmjopen.bmj.com/site/about/guidelines.xhtml

be assumed to be missing at random conditional upon other covariates.[20] Hence the missing values will be imputed using the multiple imputation by chained equations (MACE).[21] To improve precision of the imputation, the existing qualitative information from Read codes stating the direction of change (e.g. "Weight increasing (1622.00)") or BMI status (e.g. "Overweight (22AA.00)") together with other demographic and clinical covariate data will be entered into the imputation regression model.

Additionally, in order to allow for the fact that patients may die, and their weight trajectories will be subject to informative dropout due to death, we will model weight changes using a flexible joint modelling approach.[22] Effectively, the analysis of weight history will combine three techniques: (i) complete-case analysis (including only patients with at least one, either quantitative or qualitative, weight status code in each of the phases); (ii) imputation of weight where qualitative codes are present; (iii) joint modelling to incorporate the informative missingness due to death. To check the sensitivity of the correctly specified imputation model, a fully Bayesian approach that jointly imputes missing values and estimates the parameters of the longitudinal model will also be conducted.[23-25] Weight in all three treatment groups will be modelled simultaneously; the ATD group will be used as the reference group. Any marginal pairwise comparisons will be corrected for multiple testing by applying Bonferroni correction.

Further, the sensitivity to death and to missed treatment coding will be checked. During our meetings with the patients, we were informed that weight changes are important but are not of the utmost priority. In the face of death or any serious illness, weight maintenance loses its importance. Hence, our interest in weight changes in an immortal cohort and thus we would like to model weight in patients in such health that allows them to survive to the end of the study. Patients who died during the study will be excluded.

Prevalence of obesity

Post-treatment obesity in patients will be defined as ≥30 kg/m^2, in line with the WHO definition,[26] or identified from the Read codes when quantitative values are not available. The prevalence of obesity will be assessed in the entire cohort and stratified by the treatment group.

The post-treatment BMI will be compared with those in the background population (Health Survey for England). Two measures of effect will be investigated: (i) difference in overall BMI (kg/m^2) and (ii) difference in proportions of obesity. The effects will be tested with linear regression and logistic regression, respectively, in reference to the background population. The analyses will be adjusted for age and stratified by sex. Only complete case analysis will be conducted.

Cardio-metabolic risks and mortality

The descriptive analysis of primary and secondary outcomes will provide useful summaries about the sample and the outcome measures. Besides the unadjusted descriptive statistics, such as the incidence rate of each outcome, simple graphics analysis will be provided.

Causal inference in the analysis of cardio-metabolic outcomes and mortality will be undertaken using inverse propensity weighting (IPW). This method was empirically shown to be superior to other propensity score-based approaches in analysis of multiple treatments with a binary outcome.[27] The propensity-score matching, even though well valuated in two-arm treatment, in multiple treatments might introduce bias; the inverse propensity weighting (IPW) is more efficient in such scenario. Additionally IPW method is more flexible and requires weaker unconfoundedness assumption.[28]

For peer review only - http://bmjopen.bmj.com/site/about/guidelines.xhtml

Generalised propensity weights with treatment category as dependent variable will be calculated in multinominal logistic model.[29] The model will be developed using the following covariates: age at the index date, sex, baseline serum fT4, aetiology of hyperthyroidism, smoking status, IMD, cumulative time on ATD between diagnosis and the index date, time since diagnosis, BMI status.

Variables related to treatment, as an outcome, at $p<0.10$ will be selected for inclusion in the multiple propensity weights.[30] To identify these variables, we will conduct several regression analyses with the treatment as a dependent and each potential confounder as an independent variable. Furthermore, it will be investigated whether adding interaction terms or higher-order terms for continuous variables will improve the balance of the model. The Hausman test will be used to check the Independence of Irrelevant Alternatives (IIA) Assumption, which is the main assumption of multinomial regression analysis.[30] If this assumption is not met, multinomial probit analysis will be used instead.

The estimation of propensity weights will be conducted by the method proposed by McCaffrey.[31,32] The appropriateness of IPW analysis will be assessed by checking the overlap (positivity assumption). According to the positivity assumption, each patient should have a non-zero probability of being indicated to each treatment category. Lack of overlap in the distribution of observed pre-treatment characteristics between groups receiving different treatment indicates the positivity assumption is violated. This overlap will be checked visually. If poor overlap is identified, we will use Rubin's trimming method,[33] allowing discarding not-overlapping cases.

Further, pairwise balance in the distribution of all included variables between the three treatment groups will be tested with linear regression for continuous variables, logistic regression for binary, and multinomial logistic regression for nominal variables. The analysis will be done without and with adjustments. The propensity weights will be considered balanced if there are no statistically significant ($p<0.05$) differences between the likelihood of receiving a different type of treatment. If imbalances remain after weighting, a doubly robust estimation approach will be applied,[32] i.e. the imbalanced variables will be added to the model.

Whilst IPW will adjust for confounding at baseline, other time-varying adjustment will be applied to correct for the events between the baseline and the outcome. The proposed covariates are treatment with levothyroxine for developed hypothyroidism, log TSH levels and informative observations of number of GP visits in a unit of time.

Incidence rate ratios of outcomes will be calculated with Poisson regression and modelled adjusting for time-varying covariates as listed below. Time-to-event analysis with adjustments will be modelled. The appropriate model will be applied depending on the proportional hazard assumption's results: if the assumption holds – Cox proportional hazard regression will be used; if it is violated, alternative techniques such as time-varying measures in extended Cox models will be applied.

Proposed time-varying adjustments for both analyses will include post-index date data, i.e. treatment with levothyroxine for developed hypothyroidism, logTSH levels, and informative observations of number of GP visits in a unit of time. To allow for the fact that patients may die, a competing risks approach will be adopted for estimating comparative incidences for non-fatal events to allow for the impact of mortality as a competing risk.[22]

For peer review only - http://bmjopen.bmj.com/site/about/guidelines.xhtml

Sensitivity to missed radioiodine record

Whenever ATD patients develop permanent hypothyroidism, a radioiodine treatment will be assumed, as explained in the Exposure section. All analyses will be repeated to check sensitivity to this assumption, excluding patients in the ATD group who developed hypothyroidism following medical treatment being the only antithyroid treatment on record.

PATIENT AND PUBLIC INVOLVEMENT

Our patients with hyperthyroidism have been pivotal in formulating the research questions and informing the study design. The study idea originated from listening to patients and their concerns. As part of the consultation process, we held a meeting, advertised locally and nationally via the British Thyroid Foundation (BTF), with patients who had either previously been treated or were currently being treated for hyperthyroidism. During an open discussion, key themes were identified as important to patients across the treatment course of hyperthyroidism. At diagnosis, patients often reported that there was insufficient information to determine which treatment choice would be best for them, particularly regarding long-term treatment consequences; hence, a major driver of the study design is the examination of long term cardio-metabolic outcomes. The comment that "radioactive iodine will make me fat" was repeatedly mentioned in the group discussion. We were already interested in the potential issue of weight gain beyond premorbid weight, and this is something we will be investigating in this project. The main themes reported were that in addition to insufficient information to make a clear, informed treatment choice, patients did not feel that they were suitably counselled about weight regain nor that there was a mechanism in place to predict or modulate this potential effect. The insufficiency of information on the treatment choices and their long-term consequences was further confirmed in a BTF survey involving 353 patients. Among responders who received definitive treatment (n=167) who therefore had knowledge of post-definitive treatment procedure effects, a third would have not decided to proceed with their choice of treatment.

To continue guidance by patients, we have formed a patient group, which was engaged at the design stage and will continue to advise us during the project. Additionally, a BTF representative (JP) joined our team as a co-applicant to represent patients' voices regularly throughout the research process. As the study progresses, the patient group and the patients' representative will be involved in rationalising the design if any challenges are encountered, in discussing the implications and relevance of the emerging results and in finalising the key messages of the study to facilitate patient-centred dissemination. We will involve our patient partners in the dissemination of the results and the development of future avenues of research.

Additional guidance comes from the GP advisory group (SF, PS), who have shared their experience and expertise from the front-end of data collection. This was especially important while establishing the feasibility of modelling and interpreting the weight changes based on the already recorded weight entries. The GP advice will be further utilised in the interpretation of results, dissemination process and the planning of further initiatives.

ETHICS AND DISSEMINATION

For peer review only - http://bmjopen.bmj.com/site/about/guidelines.xhtml

The study protocol has been approved by the Independent Scientific Advisory Committee (ISAC) in March 2021 (#20_000185). ISAC is a non-statutory expert advisory body established in 2006 by the Secretary of State for Health to provide scientific advice on research requests to access data provided by CPRD.

The primary aim is to inform clinical practice within the NHS and provide evidence for the future research in managing long-term consequences of hyperthyroidism and its treatment. The dissemination plan targets audiences at various levels: clinical and academic as well as patients and public. To address the needs of the former, we are planning to share the findings at the scientific endocrinology meetings and to publish them in leading open-access peer-reviewed academic journals. The British Thyroid Foundation (BTF), a national community of patients with thyroid conditions in the UK, will play a key role in disseminating the results of the study to the public, patients and their families and carers. The BTF will share results via traditional and social media channels.

For peer review only

CONTRIBUTION:

BT, JMH, KN, GNT, KRA and KB participated in the conception and the initial design of the study, with further substantial contributions from SF, PS and JP. All authors participated in drafting the protocol and have approved the final version.

ACKNOWLEDGEMENT:

The authors would like to thank Prof. Daniel Lasserson for special contribution to the study protocol.

FUNDING:

This project is funded by the National Institute for Health Research (NIHR) under its Research for Patient Benefit (RfPB) Programme (Grant Number: NIHR200772). The views expressed are those of the authors and not necessarily those of the NIHR or the Department of Health and Social Care.

COMPETING INTERESTS:

BT, JMH, KN, GNT, JP, SF, PS, KB have nothing to declare.

KRA has served as a paid consultant, providing unrelated methodological advice to: Abbvie, Amaris, Allergan, Astellas, AstraZeneca, Boehringer Ingelheim, Bristol-Meyers Squibb, Creativ-Ceutical, GSK, ICON/Oxford Outcomes, Ipsen, Janssen, Eli Lilly, Merck, NICE, Novartis, NovoNordisk, Pfizer, PRMA, Roche and Takeda, and has received research funding from Association of the British Pharmaceutical Industry (ABPI), European Federation of Pharmaceutical Industries & Associations (EFPIA), Pfizer and Sanofi. He is a Partner and Director of Visible Analytics Limited, a healthcare consultancy company.

For peer review only - http://bmjopen.bmj.com/site/about/guidelines.xhtml

REFERENCES

1. Vanderpump MP. The epidemiology of thyroid disease. Br Med Bull. 2011;99:39-51.
2. Boelaert K, Torlinska B, Holder RL, Franklyn JA. Older subjects with hyperthyroidism present with a paucity of symptoms and signs: a large cross-sectional study. J Clin Endocrinol Metab. 2010;95(6):2715-26.
3. Brandt F, Green A, Hegedus L, Brix TH. A critical review and meta-analysis of the association between overt hyperthyroidism and mortality. Eur J Endocrinol. 2011;165(4):491-7.
4. Abraham P, Avenell A, McGeoch SC, Clark LF, Bevan JS. Antithyroid drug regimen for treating Graves' hyperthyroidism. Cochrane Database Syst Rev. 2010(1):CD003420.
5. Ross DS, Burch HB, Cooper DS, Greenlee MC, Laurberg P, Maia AL, et al. 2016 American Thyroid Association Guidelines for Diagnosis and Management of Hyperthyroidism and Other Causes of Thyrotoxicosis. Thyroid. 2016;26(10):1343-421.
6. NICE. Hyperthyroidism.: National Institute for Health and Care Excellence; 2020 [13/04/2021]. Available from: https://cks.nice.org.uk/topics/hyperthyroidism/.
7. Boelaert K, Maisonneuve P, Torlinska B, Franklyn JA. Comparison of mortality in hyperthyroidism during periods of treatment with thionamides and after radioiodine. J Clin Endocrinol Metab. 2013;98(5):1869-82.
8. Ryodi E, Metso S, Huhtala H, Valimaki M, Auvinen A, Jaatinen P. Cardiovascular Morbidity and Mortality After Treatment of Hyperthyroidism with Either Radioactive Iodine or Thyroidectomy. Thyroid. 2018;28(9):1111-20.
9. Dale J, Daykin J, Holder R, Sheppard MC, Franklyn JA. Weight gain following treatment of hyperthyroidism. Clin Endocrinol (Oxf). 2001;55(2):233-9.
10. Rotondi M, Croce L, Pallavicini C, Manna LL, Accornero SM, Fonte R, et al. Body weight changes in a large cohort of patients subjected to thyroidectomy for a wide spectrum of thyroid diseases. Endocr Pract. 2014;20(11):1151-8.
11. Torlinska B, Nichols L, Mohammed MA, McCabe C, Boelaert K. Patients Treated for Hyperthyroidism Are at Increased Risk of Becoming Obese: Findings from a Large Prospective Secondary Care Cohort. Thyroid. 2019;29(10):1380-9.
12. Galindo RJ, Hurtado CR, Pasquel FJ, Garcia Tome R, Peng L, Umpierrez GE. National Trends in Incidence, Mortality, and Clinical Outcomes of Patients Hospitalized for Thyrotoxicosis With and Without Thyroid Storm in the United States, 2004-2013. Thyroid. 2019;29(1):36-43.
13. Metso S, Auvinen A, Salmi J, Huhtala H, Jaatinen P. Increased long-term cardiovascular morbidity among patients treated with radioactive iodine for hyperthyroidism. Clin Endocrinol (Oxf). 2008;68(3):450-7.
14. Okosieme OE, Taylor PN, Evans C, Thayer D, Chai A, Khan I, et al. Primary therapy of Graves' disease and cardiovascular morbidity and mortality: a linked-record cohort study. Lancet Diabetes Endocrinol. 2019;7(4):278-87.
15. Selmer C, Olesen JB, Hansen ML, Lindhardsen J, Olsen AMS, Madsen JC, et al. The spectrum of thyroid disease and risk of new onset atrial fibrillation: a large population cohort study. Bmj-British Medical Journal. 2012;345:12.
16. Baumgartner C, da Costa BR, Collet TH, Feller M, Floriani C, Bauer DC, et al. Thyroid Function Within the Normal Range, Subclinical Hypothyroidism, and the Risk of Atrial Fibrillation. Circulation. 2017;136(22):2100-16.
17. Gencer B, Collet TH, Virgini V, Bauer DC, Gussekloo J, Cappola AR, et al. Subclinical thyroid dysfunction and the risk of heart failure events: an individual participant data analysis from 6 prospective cohorts. Circulation. 2012;126(9):1040-9.
18. Thayakaran R, Adderley NJ, Sainsbury C, Torlinska B, Boelaert K, Sumilo D, et al. Thyroid replacement therapy, thyroid stimulating hormone concentrations, and long term health outcomes in patients with hypothyroidism: longitudinal study. BMJ. 2019;366:l4892.

For peer review only - http://bmjopen.bmj.com/site/about/guidelines.xhtml

19. Herrett E, Gallagher AM, Bhaskaran K, Forbes H, Mathur R, van Staa T, et al. Data Resource Profile: Clinical Practice Research Datalink (CPRD). Int J Epidemiol. 2015;44(3):827-36.
20. Marston L, Carpenter JR, Walters KR, Morris RW, Nazareth I, Petersen I. Issues in multiple imputation of missing data for large general practice clinical databases. Pharmacoepidemiol Drug Saf. 2010;19(6):618-26.
21. Royston P, White IR. Multiple Imputation by Chained Equations (MICE): Implementation in Stata. Journal of Statistical Software. 2011;45(4):1-20.
22. Crowther MJ, Abrams KR, Lambert PC. Joint Modeling of Longitudinal and Survival Data. The Stata Journal. 2013;13(1):165-84.
23. Erler NS, Rizopoulos D, Jaddoe VW, Franco OH, Lesaffre EM. Bayesian imputation of time-varying covariates in linear mixed models. Stat Methods Med Res. 2019;28(2):555-68.
24. Erler NS, Rizopoulos D, Lesaffre EM. JointAI: Joint Analysis and Imputation of Incomplete Data in R. arXiv [Internet]. 2020 20/06/2021. Available from: https://arxiv.org/abs/1907.10867.
25. Erler NS, Rizopoulos D, Rosmalen Jv, Jaddoe VWV, Franco OH, Lesaffre EMEH. Dealing with missing covariates in epidemiologic studies: a comparison between multiple imputation and a full Bayesian approach. Statistics in Medicine. 2016;35(17):2955-74.
26. World Health Organisation. Body mass index - BMI. 2021 [30/03/2021]. Available from: https://www.euro.who.int/en/health-topics/disease-prevention/nutrition/a-healthy-lifestyle/body-mass-index-bmi.
27. Yu Y, Zhang M, Shi X, Caram MEV, Little RJA, Mukherjee B. A comparison of parametric propensity score-based methods for causal inference with multiple treatments and a binary outcome. Statistics in Medicine. 2021;40(7):1653-77.
28. Lopez MJ, Gutman R. Estimation of Causal Effects with Multiple Treatments: A Review and New Ideas. Statistical Science. 2017;32(3):432-54.
29. Imbens G. The role of the propensity score in estimating dose-response functions. Biometrika. 2000;87(3):706-10.
30. Spreeuwenberg MD, Bartak A, Croon MA, Hagenaars JA, Busschbach JJV, Andrea H, et al. The Multiple Propensity Score as Control for Bias in the Comparison of More Than Two Treatment Arms: An Introduction From a Case Study in Mental Health. Medical Care. 2010;48(2):166-74.
31. Cefalu M, Buenaventura M. Propensity Scores for Multiple Treatments: A Tutorial on the MNPS Command for Stata Users: RAND Corporation; 2017.
32. McCaffrey DF, Griffin BA, Almirall D, Slaughter ME, Ramchand R, Burgette LF. A tutorial on propensity score estimation for multiple treatments using generalized boosted models. Stat Med. 2013;32(19):3388-414.
33. Rubin DB. Estimating causal effects of treatments in randomized and nonrandomized studies. Journal of Educational Psychology. 1974;66(5):688-701.

For peer review only

For peer review only - http://bmjopen.bmj.com/site/about/guidelines.xhtml

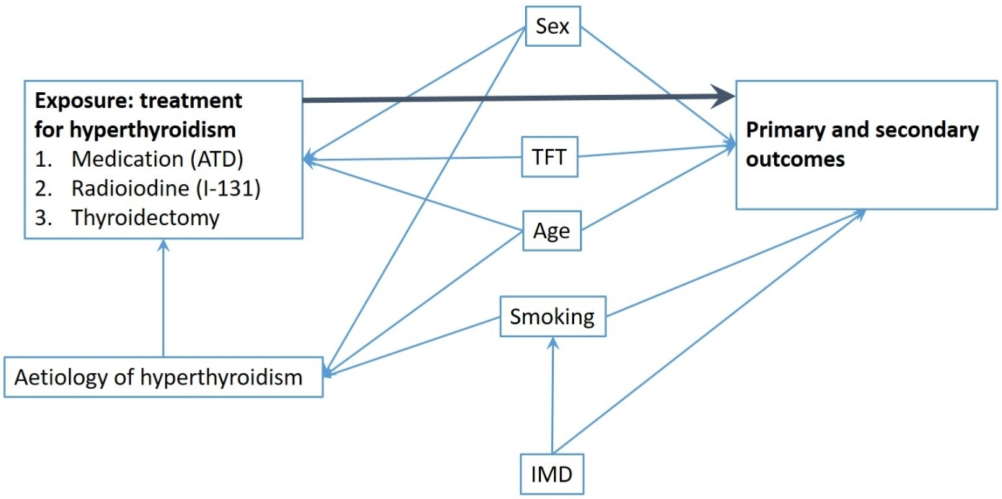

Figure 1: Directed acyclic graph (DAG) illustrating confounding in the study. The wide dark arrow indicates the relationship of interest. ATD: antithyroid drugs, TFT: thyroid function test, IMD: Index of Multiple Deprivation

259x130mm (150 x 150 DPI)

APPENDIX: Medical codes used for data search

Table 1: Read codes for diagnosis of hyperthyroidism

Read code	Description
C02..00	Thyrotoxicosis
C02..11	Hyperthyroidism
C02..12	Toxic goitre
C020.00	Toxic diffuse goitre
C020.11	Basedow's disease
C020.12	Graves' disease
C020000	Toxic diffuse goitre with no crisis
C020100	Toxic diffuse goitre with crisis
C020200	Thyroid-associated dermopathy
C020z00	Toxic diffuse goitre NOS
C021.00	Toxic uninodular goitre
C021000	Toxic uninodular goitre with no crisis
C021100	Toxic uninodular goitre with crisis
C021z00	Toxic uninodular goitre NOS
C022.00	Toxic multinodular goitre
C022000	Toxic multinodular goitre with no crisis
C022100	Toxic multinodular goitre with crisis
C022z00	Toxic multinodular goitre NOS
C023.00	Toxic nodular goitre unspecified
C023000	Toxic nodular goitre unspecified with no crisis
C023100	Toxic nodular goitre unspecified with crisis
C023z00	Toxic nodular goitre NOS
C024.00	Thyrotoxicosis from ectopic thyroid nodule
C024000	Thyrotoxicosis from ectopic thyroid nodule with no crisis
C024100	Thyrotoxicosis from ectopic thyroid nodule with crisis
C024z00	Thyrotoxicosis from ectopic thyroid nodule NOS
C02y.00	Thyrotoxicosis of other specified origin
C02y.11	Factitia thyrotoxicosis
C02y000	Thyrotoxicosis of other specified origin with no crisis
C02y100	Thyrotoxicosis of other specified origin with crisis
C02y200	Thyrotoxicosis factitia
C02y300	Thyroid crisis
C02yz00	Thyrotoxicosis of other specified origin NOS

For peer review only

C02z.00	Thyrotoxicosis without mention of goitre or other cause
C02z000	Thyrotoxicosis without mention of goitre or cause no crisis
C02z100	Thyrotoxicosis without mention of goitre, cause with crisis
C02zz00	Thyrotoxicosis NOS

Table 2: Codes for the treatment group allocation

Treatment group allocation	Coding system		
	Read	BNF	OPCS-4
**Medication:**			
Carbimazole		06.02.02.00	
Propylthiouracil		06.02.02.00	
**Radioiodine:**			
Radioactive drug therapy	5A16.00		X65.5
I131 radiotherapy	5A16.11		
Iodine 131 radiotherapy	5A16.12		
**Thyroidectomy:**			
Thyroid gland operations	711..12		B12.8
Thyroidectomy operations	7110.00		
Excision of thyroid gland operations	7110.11		
Total thyroidectomy	7110000		B08.1
Subtotal thyroidectomy	7110100		B08.2
Bilateral subtotal thyroidectomy	7110111		B08.2
Hemithyroidectomy	7110200		B08.3
Lobectomy of thyroid gland NEC	7110300		B08.4
Isthmectomy of thyroid gland	7110400		B08.5
Partial thyroidectomy NEC	7110500		B08.6
Thyroidectomy NEC	7110600		B08.6
Other specified thyroidectomy	7110y00		B08.8
Thyroidectomy NOS	7110z00		B08.9

For peer review only

Table 3: Outcomes codes

Outcome	Coding system	
	**ICD-10**	**Read codes**
obesity		22K5.00, 66C..00, 66CZ.00, C38..00, C380.00, C380.00, C380000, C380100, C380200, C380300, C380400, C380500, C38y011, C38z.00, C38z000
nonfatal myocardial infarction (composite of MACE)	I21-I22	n/a
nonfatal stroke (composite of MACE)	I64	n/a
congestive heart failure	I50	n/a
ischaemic heart disease	I20-I25	n/a
stroke and transient ischaemic attack (TIA)	I60-I69	n/a
type 2 diabetes mellitus	E11	C109*

MACE: major adverse cardiovascular event; TIA: transient ischemic attack

For peer review only

BMJ Open

wEight chanGes, caRdio-mEtabolic risks and morTality in patients with hyperthyroidism (EGRET): a protocol for a CPRD–HES linked cohort study

Journal:	*BMJ Open*
Manuscript ID	bmjopen-2021-055219.R1
Article Type:	Protocol
Date Submitted by the Author:	06-Sep-2021
Complete List of Authors:	Torlinska, Barbara; University of Birmingham, Institute of Applied Health Research; University Hospitals Birmingham NHS Foundation Trust, NIHR Birmingham Biomedical Research Centre<br>Hazlehurst, Jonathan; University of Birmingham, Institute of Applied Health Research; University Hospitals Birmingham NHS Foundation Trust, Department of Diabetes and Endocrinology<br>Nirantharakumar, Krishnarajah; University of Birmingham, Institute of Applied Health Research; University of Birmingham, Midlands Health Data Research UK<br>Thomas, Neil; University of Birmingham, Institute of Applied Health Research<br>Priestley, Julia; British Thyroid Foundation<br>Finnikin, Samuel; University of Birmingham, Institute of Applied Health Research<br>Saunders, Philip; Ridgacre Medical Centres<br>Abrams, Keith; University of Warwick, Department of Statistics; University of York, Centre for Health Economics<br>Boelaert, K; University of Birmingham, Institute of Applied Health Research; University Hospitals Birmingham NHS Foundation Trust, Birmingham Health Partners
**Primary Subject Heading**:	Diabetes and endocrinology
Secondary Subject Heading:	Epidemiology, Cardiovascular medicine
Keywords:	Thyroid disease < DIABETES & ENDOCRINOLOGY, Cardiac Epidemiology < CARDIOLOGY, EPIDEMIOLOGY

SCHOLARONE™
Manuscripts

I, the Submitting Author has the right to grant and does grant on behalf of all authors of the Work (as defined in the below author licence), an exclusive licence and/or a non-exclusive licence for contributions from authors who are: i) UK Crown employees; ii) where BMJ has agreed a CC-BY licence shall apply, and/or iii) in accordance with the terms applicable for US Federal Government officers or employees acting as part of their official duties; on a worldwide, perpetual, irrevocable, royalty-free basis to BMJ Publishing Group Ltd ("BMJ") its licensees and where the relevant Journal is co-owned by BMJ to the co-owners of the Journal, to publish the Work in this journal and any other BMJ products and to exploit all rights, as set out in our licence.

The Submitting Author accepts and understands that any supply made under these terms is made by BMJ to the Submitting Author unless you are acting as an employee on behalf of your employer or a postgraduate student of an affiliated institution which is paying any applicable article publishing charge ("APC") for Open Access articles. Where the Submitting Author wishes to make the Work available on an Open Access basis (and intends to pay the relevant APC), the terms of reuse of such Open Access shall be governed by a Creative Commons licence – details of these licences and which Creative Commons licence will apply to this Work are set out in our licence referred to above.

Other than as permitted in any relevant BMJ Author's Self Archiving Policies, I confirm this Work has not been accepted for publication elsewhere, is not being considered for publication elsewhere and does not duplicate material already published. I confirm all authors consent to publication of this Work and authorise the granting of this licence.

For peer review only

wEight chanGes, caRdio-mEtabolic risks and morTality in patients with hyperthyroidism (EGRET): a protocol for a CPRD–HES linked cohort study

Barbara Torlinska,[1,2] Jonathan M. Hazlehurst,[1,3,4] Krishnarajah Nirantharakumar,[1,5] G. Neil Thomas,[1] Julia Priestley,[6] Samuel J. Finnikin,[1] Philip Saunders,[7] Keith R. Abrams*,[8,9] Kristien Boelaert*[1,3]

* joint senior authors

[1] Institute of Applied Health Research, University of Birmingham, Birmingham, UK
[2] NIHR Birmingham Biomedical Research Centre, University Hospitals Birmingham NHS Foundation Trust and University of Birmingham, Birmingham, UK
[3] Centre for Endocrinology, Diabetes and Metabolism, Birmingham Health Partners, Birmingham, UK
[4] Department of Diabetes and Endocrinology, University Hospitals Birmingham NHS Foundation Trust, Birmingham, UK
[5] Midlands Health Data Research UK, University of Birmingham, Birmingham, UK
[6] British Thyroid Foundation, Harrogate, UK
[7] Ridgacre Medical Centres, Birmingham, UK
[8] Department of Statistics, University of Warwick, Coventry, UK
[9] Centre for Health Economics, University of York, York, UK

Corresponding author:

Dr Barbara Torlinska
B.Torlinska@bham.ac.uk

Keywords: Thyrotoxicosis, Body weight, Obesity, Treatment for hyperthyroidism, Cardiometabolic outcomes, Mortality

For peer review only - http://bmjopen.bmj.com/site/about/guidelines.xhtml

ABSTRACT

Introduction: Hyperthyroidism is a common condition affecting up to 3% of the UK population. Treatment improves symptoms and reduces the risk of atrial fibrillation and stroke that contribute to increased mortality. The most common symptom is weight loss, which is reversed during treatment. However, the weight regain may be excessive, contributing to increased risk of obesity.

Current treatment options include antithyroid drugs, radioiodine and thyroidectomy. Whether there are differences in either weight change or the long-term cardio-metabolic risk between the three treatments is unclear.

Methods and analysis: The study will establish the natural history of weight change in hyperthyroidism, investigate the risk of obesity and risks of cardio-metabolic conditions and death relative to the treatment. The data on patients diagnosed with hyperthyroidism between 01/01/1996 and 31/12/2015 will come from Clinical Practice Research Datalink (CPRD) linked to Hospital Episode Statistics (HES) and ONS Death Registry.

The weight changes will be modelled using a flexible joint modelling, accounting for mortality. Obesity prevalence in the general population will be sourced from Health Survey for England and compared with the post-treatment prevalence of obesity in patients with hyperthyroidism. The incidence and time-to-event of major adverse cardiovascular events (MACE), other cardio-metabolic outcomes and mortality will be compared between the treatments using the inverse propensity weighting model. Incidence rate ratios of outcomes will be modelled with Poisson regression. Time-to-event will be analysed using Cox proportional hazards model. A competing risks approach will be adopted to estimate comparative incidences to allow for the impact of mortality.

Ethics and dissemination: The study will bring new knowledge on the risk of developing obesity, cardio-metabolic morbidity and mortality following treatment for hyperthyroidism to inform the clinical practice and public health policies. The results will be disseminated via open-access peer-reviewed publications and directly to the patients and public groups. (ISAC protocol approval #20_000185)

STRENGTHS AND LIMITATIONS OF THIS STUDY:

- Rich data from a large sample allowing for pre-exposure history and long follow-up, spanning across primary and secondary care
- Contextualisation of post-treatment obesity prevalence by comparison with general population adjusted for sex and age
- A broad range of patient relevant cardio-metabolic outcomes will be evaluated
- Large real world observational study of treatment options not amenable to randomised clinical trials
- The main study's limitations are related to the intrinsic nature of the real-world data, such as the secondary use of data or the presence of missing data

INTRODUCTION

Hyperthyroidism is a common condition affecting 3% of women and 0.3% of men in the UK.[1] Common clinical features indicating hyperthyroidism include weight loss, heat intolerance, tremor,

For peer review only

palpitations and anxiety.[2] Treatment is critical to minimise complications including atrial fibrillation and stroke that contribute to the observed 20% increase in mortality.[3]

There are three treatment options: antithyroid drugs (ATDs), radioactive iodine (I-131, radioiodine), or surgical either as a total or hemi-thyroidectomy. ATDs are associated with a high rate of relapse (30-70%);[4] while treatment with radioiodine or thyroidectomy leads to the development of hypothyroidism, requiring life-long levothyroxine replacement, which is seen in 80% of patients administered radioiodine and up to 100% undergoing thyroidectomy. Guidance recommends discussion of all three options with the patient.[5,6] There is insufficient evidence to recommend one treatment over the others, although some studies indicate differences in cardiac events and mortality between the treatment modalities.[7,8]

Weight gain following various treatments for hyperthyroidism has been described.[9,10] Clinicians commonly assume that the observed weight gain is a simple regain of weight lost prior to the initiation of treatment. However, an analysis of 1,373 patients with hyperthyroidism has demonstrated a higher prevalence of obesity at three years of follow-up when compared with the age and sex matched background population (37% vs 26% in men, p<0.001; 32% vs 26% in women, p<0.001).[11] These findings suggest excess weight gain beyond patients' premorbid weight (weight overshoot). Additionally, the study showed significant differences in final weight gain compared to baseline between those treated with ATDs (5.4 kg [95%CI: 4.8–6.0) and those who received radioiodine but did not develop hypothyroidism (5.1 kg [4.3–6.1]) when compared to those treated with radioiodine who developed subsequent hypothyroidism (7.1 kg [6.6–7.7]). The extent of weight change in patients treated with thyroidectomy was not evaluated.

Prior to diagnosis, the majority of patients with hyperthyroidism notice that they lose weight, often despite increased appetite, due to the regulatory effects of thyroid hormones on metabolism. As thyroid hormone levels normalise following treatment, weight change may be observed. While weight gain following treatment is common, it may become a major psychological stressor and can affect treatment compliance. Untreated hyperthyroidism may result in serious consequences, including thyroid storm, heart failure, embolic events, atrial fibrillation, osteoporosis, muscle weakness, neuropsychiatric symptoms, and rarely cardiovascular collapse and death.[12] Patients need to be appropriately counselled about the possibility of significant weight gain associated with the treatment of hyperthyroidism. However, there is currently insufficient data available about weight overshoot, cardiovascular risk and treatment modality selection to provide clear guidance to our patients. In our recent survey of British Thyroid Association members, of 35 clinicians that responded, only 23% usually discuss weight gain with patients treated for hyperthyroidism despite 70% of respondents feeling this was a significant clinical problem.

We recently surveyed doctors and dieticians and found that whilst there was agreement that weight gain following treatment for hyperthyroidism represents a medical problem, there is no consensus about the significance of or the approach to this anticipated weight gain. When surveyed, dieticians expressed concerns that they are not equipped to advise on dietary issues given the switch from a highly catabolic state (weight loss state) in untreated hyperthyroidism to a situation of anticipated weight gain following treatment.

As previously shown, hyperthyroidism is associated with increased cardiovascular morbidity and mortality, which is not completely reversed by treatment.[7,8,13,14] Among cardiovascular comorbidities, atrial fibrillation is the most common, occurring in 2 to 20% of untreated patients[15] and complications including heart failure and thrombo-embolic events are observed in patients with hyperthyroidism. In a recent meta-analysis, an increased risk of atrial fibrillation with higher

For peer review only - http://bmjopen.bmj.com/site/about/guidelines.xhtml

concentrations of thyroid hormones at presentation was confirmed.[16] In a nation-wide register study with a mean follow-up time of 10 years, an increased risk of all arrhythmias was found in patients undergoing treatment with radioiodine or thyroidectomy when compared with controls with no thyroid dysfunction.[8] Patients with subclinical hyper- and hypothyroid dysfunction were found to have increased risks of heart failure (HR=1.94 [95%CI 1.01– 3.72] for TSH <0.10 mIU/L and 1.86 [1.27–2.72] for TSH >10.0 mIU/L) in a pooled analysis of individual participant data using available prospective cohorts with thyroid function tests and subsequent follow-up of heart failure.[17] In a large cohort of UK patients on levothyroxine replacement, mortality was increased in the lowest (<0.1) and highest TSH categories (>4.0) compared with 2-2.5 mIU/L, while risks of ischaemic heart disease and heart failure were found to increase at high concentrations of TSH (>10.0 mIU/L).[18]

There is insufficient evidence to systematically review the differences in health outcomes between the three different treatments. Due to limitations arising from the need to adhere to radiation protection and varied requirements of the procedures (a prolonged course of medication, one-off radioiodine tablet or surgical procedure), randomisation and blinding between all three modalities is often not possible, making randomised controlled trials nearly non-existent in this area of research.

At our recent patient engagement event, patients felt that there was insufficient information to help guide the choice of treatment. They were concerned about the risk of excessive weight gain and its long-term cardio-metabolic consequences and frustrated with the lack of information, allowing them to prioritise one treatment over the other. There was also disappointment about the lack of support services addressing weight and diet across treatment for hyperthyroidism. Patients were particularly concerned about the perceived risk of weight gain associated with radioactive iodine and agreed that proper quantification of weight gain and cardiovascular risks across the treatments would influence their therapy preferences and could inform anticipatory dietary modification.

For peer review only

Aims and objectives:

The overall aim of the study is to investigate the risks of gaining weight and developing obesity, developing cardio-metabolic conditions or death following treatment for hyperthyroidism and to compare these risks between the three treatment modalities.

Primary objective

The primary aim of this study is to assess the effect of type of treatment on weight changes during and after therapy for hyperthyroidism. The post-treatment prevalence of obesity will also be evaluated and compared with that of the general population.

Secondary objectives

Further, the effect of treatment and weight status on the incidence of major cardiovascular events (MACE), other cardio-metabolic outcomes and mortality following each treatment modality will be evaluated.

For peer review only - http://bmjopen.bmj.com/site/about/guidelines.xhtml

METHODS

This is a retrospective, longitudinal, observational study using routinely collected data. Data for the study will come from the Clinical Practice Research Datalink (CPRD), linked to Hospital Episode Statistics (HES) admissions and outpatients databases. The study is funded by the National Institute of Health Research (NIHR, and is to be conducted between 01/02/2021 and 31/07/2022. The protocol was approved by the Independent Scientific Advisory Committee (ISAC #20_000185).

Data sources

CPRD collects and anonymises patient electronic health record data from a network of GP practices across the UK.[19] Although the records in the database cover all four UK countries, HES-linkable records are limited to those from England only. Two HES datasets will be used: HES outpatient and HES admitted patient care. Linkage to HES databases is required to obtain the data, which may be missing from the GP records on non-medical treatment for hyperthyroidism (radioactive iodine and thyroidectomy). Additionally, data on major cardiovascular events (MACE) will also be sourced from HES, as such events are typically being treated in the hospital setting.

Linkage to social data is required to adjust the analysis for socioeconomic status, which is a known risk factor for obesity and increased mortality. This will be obtained as the Index of Multiple Deprivation (IMD). Linkage to Office of National Statistics (ONS) data is required to get access to the date of death and cause of death. Prevalence of post-treatment obesity will be compared with the background population sourced from Health Survey for England, a yearly survey monitoring trends in the nation's health and care.

Population

Adult patients (≥18 years) who were for the first time diagnosed with hyperthyroidism (the list of codes in Appendix) between 01/01/1996 and 31/12/2015 will be eligible for inclusion. To minimise missed cases, the diagnosis will be additionally identified by a prescription of carbimazole or polythiurauracil, which are antithyroid drugs indicated only for treatment of hyperthyroidism. A minimum of 12 months of data must be available before the date of diagnosis and after the index date. CPRD records need to be classified as acceptable research quality.

Exclusion criteria encompass antithyroid drug treatment of duration shorter than six months as the only treatment, i.e. with no radioactive iodine or thyroidectomy followed. This is to avoid contamination by misdiagnosis or spontaneously resolving thyroiditis. Patients treated with both definitive treatment methods, i.e. radioiodine and thyroidectomy, will also be excluded. However, ATD treatment followed by either radioiodine or thyroidectomy (one of the definitive treatments) is considered a pre-treatment and does not constitute an exclusion criterion.

Exposure

The exposure in the study is the treatment for hyperthyroidism The study patients will be divided into three groups based on the treatment administered: (i) Radioiodine: The allocation to this treatment arm will be assigned based on the Read code or OPCS-4 code of the treatment procedure for radioiodine; (ii) Thyroidectomy: The allocation to this treatment arm will be assigned based on

For peer review only - http://bmjopen.bmj.com/site/about/guidelines.xhtml

the Read code or OPCS-4 code of the surgical procedure; (iii) Medical treatment arm will be identified based on the absence of radioiodine treatment code and thyroidectomy code in the presence of ATD treatment (based on the Read code or BNF code) longer than 6 months.

We anticipate good quality of ATD prescription and thyroidectomy. However, radioiodine, typically administered in outpatients, may be missed in a substantially high proportion of patients. Since ATD treatment does not incur permanent hypothyroidism, the long-term prescription of levothyroxine for hypothyroidism following treatment with ATD alone, i.e. without any definitive procedures, will be considered as a proxy for radioiodine administration.

Outcomes

The primary outcome is weight change (in kg) during and following the treatment for hyperthyroidism, as well as prevalence of obesity following the treatment. The secondary outcomes include cardio-metabolic events and mortality as presented in table 1.

Table 1: Secondary outcomes and their data sources.

Secondary outcome	Source
MACE (cardiovascular death, nonfatal myocardial infarction, or nonfatal stroke)	HES
type 2 diabetes mellitus	CPRD
congestive heart failure	HES
ischaemic heart disease	HES
stroke and transient ischaemic attack	HES
cardiovascular mortality	ONS
all-cause mortality	ONS

MACE: major adverse cardiovascular events, HES: Health Episode Statistics, CPRD: Clinical Practice Research Database, ONS: Office for National Statistics

Confounding

Based on the literature and clinical experience, we identified a number of confounders affecting both the exposure and the outcomes of our study. The modelled outcomes will be adjusted for baseline data (sex, age, IMD quartiles, smoking status, comorbidities) and time-varying covariates (thyroid function, cumulative time on the medical treatment, time since diagnosis, levothyroxine replacement, pregnancy and diagnosis of cancer). Table 2 presents the details of how covariates are defined and handled. Figure 1 depicts the directed acyclic graphs representing the relationship between the covariates. We will explore the relationships between the potential confounders and both exposures and outcomes independently in order to establish their potential role in this particular dataset.

Table 2: Definitions of study covariates

Variable	Definition
Aetiology of hyperthyroidism	Identified based on Read codes and categorised in (i) Graves' disease, (ii) toxic nodular goitre, and (iii) undefined. The undefined category will consist of unspecified diagnosis and missing aetiology data.
Age	Age at index date

For peer review only - http://bmjopen.bmj.com/site/about/guidelines.xhtml

Baseline fT4	The highest measurement between 3 months prior to diagnosis and the index date
Body Mass Index (BMI)	Normal (or underweight) <25 kg/m^2, overweight 25-30 kg/m^2, and obese ≥ 30 kg/m^2; additionally BMI status will be deduced from the Read code
Cancer diagnosis	Switch-type binary variable assuming life-long status of cancer comorbidity
Frequency of GP visits	Time-varying variable of number of GP visits in a unit of time
Index of multiple deprivation (IMD)	IMD will be stratified into quintiles
Levothyroxine (LT4) replacement	Switch-type binary variable assuming life-long LT4 administration following development of hypothyroidism in Radioiodine and Thyroidectomy treatment groups
Pregnancy	Time-varying binary variable recording pregnancy
Pre-treatment ATD	Cumulative time on ATD between diagnosis and the index date
Sex	Binary variable
Smoking status	As recorded at the index date; last observation carried forward (LOCF) if not available at the index date
Thyroid Stimulating Hormone (TSH)	Serial TSH, log-transformed for the analysis
Time since diagnosis	Time-varying variable measuring cumulative time since diagnosis

Index date and follow-up

The index date in the cohort is the date of initiation of treatment for hyperthyroidism, which defines the treatment group (either ATD, radioiodine or thyroidectomy). There is a requirement of at least 12 months data available prior to the index date.
The natural history of weight changes will be modelled pre-and post-index date. While the follow-up for the analysis of outcomes will start at the index date; a minimum of 12 month follow-up is required for the inclusion.

DATA ANALYSIS

Natural history of weight changes

Descriptive statistics will provide summaries about the sample and weight as the outcome measure. The natural history of weight changes will be modelled longitudinally. The model will estimate the weight (kg) over three stages: premorbid, duration of treatment, and post-treatment. Premorbid weight is defined as body weight recorded at least 3 months before diagnosis or treatment initiation (whichever comes first). The treatment phase will start from the time of diagnosis or the first prescribed dose or procedure and will last until three months after the last recorded date of treatment. Any weight recoded thereafter will be considered as post-treatment.

Only patients having at least one weight record (quantitative or qualitative) in each of the three phases of the condition (premorbid, treatment, post-treatment) will be included in the natural

For peer review only - http://bmjopen.bmj.com/site/about/guidelines.xhtml

history of weight analysis. The extent of missing data will be reported. As with any real-world data, we anticipate a relatively high proportion of intermittent missing weight data. This missingness can be assumed to be missing at random conditional upon other covariates.[20] Hence the missing values will be imputed using the multiple imputation by chained equations (MACE).[21] To improve precision of the imputation, the existing qualitative information from Read codes stating the direction of change (e.g. "Weight increasing (1622.00)") or BMI status (e.g. "Overweight (22AA.00)") together with other demographic and clinical covariate data will be entered into the imputation regression model.

Additionally, in order to allow for the fact that patients may die, and their weight trajectories will be subject to informative dropout due to death, we will model weight changes using a flexible joint modelling approach.[22] Effectively, the analysis of weight history will combine three techniques: (i) complete-case analysis (including only patients with at least one, either quantitative or qualitative, weight status code in each of the phases); (ii) imputation of weight where qualitative codes are present; (iii) joint modelling to incorporate the informative missingness due to death. To check the sensitivity of the correctly specified imputation model, a fully Bayesian approach that jointly imputes missing values and estimates the parameters of the longitudinal model will also be conducted.[23-25] Weight in all three treatment groups will be modelled simultaneously; the ATD group will be used as the reference group. Any marginal pairwise comparisons will be corrected for multiple testing by applying Bonferroni correction.

Further, the sensitivity to death and to missed treatment coding will be checked. During our meetings with the patients, we were informed that weight changes are important but are not of the utmost priority. In the face of death or any serious illness, weight maintenance loses its importance. Hence, our interest in weight changes in an immortal cohort and thus we would like to model weight in patients in such health that allows them to survive to the end of the study. Patients who died during the study will be excluded.

Prevalence of obesity

Post-treatment obesity in patients will be defined as ≥30 kg/m^2, in line with the WHO definition,[26] or identified from the Read codes when quantitative values are not available. The prevalence of obesity will be assessed in the entire cohort and stratified by the treatment group.

The post-treatment BMI will be compared with those in the background population (Health Survey for England). Two measures of effect will be investigated: (i) difference in overall BMI (kg/m^2) and (ii) difference in proportions of obesity. The effects will be tested with linear regression and logistic regression, respectively, in reference to the background population. The analyses will be adjusted for age and stratified by sex. Only complete case analysis will be conducted.

Cardio-metabolic risks and mortality

The descriptive analysis of primary and secondary outcomes will provide useful summaries about the sample and the outcome measures. Besides the unadjusted descriptive statistics, such as the incidence rate of each outcome, simple graphics analysis will be provided.

Causal inference in the analysis of cardio-metabolic outcomes and mortality will be undertaken using inverse propensity weighting (IPW). This method was empirically shown to be superior to other propensity score-based approaches in analysis of multiple treatments with a binary outcome.[27] The propensity-score matching, even though well valuated in two-arm treatment, in multiple treatments

For peer review only - http://bmjopen.bmj.com/site/about/guidelines.xhtml

might introduce bias; the inverse propensity weighting (IPW) is more efficient in such scenario. Additionally IPW method is more flexible and requires weaker unconfoundedness assumption.[28]

Generalised propensity weights with treatment category as dependent variable will be calculated in multinominal logistic model.[29] The model will be developed using the following covariates: age at the index date, sex, baseline serum fT4, aetiology of hyperthyroidism, smoking status, IMD, cumulative time on ATD between diagnosis and the index date, time since diagnosis, BMI status.

Variables related to treatment, as an outcome, at p<0.10 will be selected for inclusion in the multiple propensity weights.[30] To identify these variables, we will conduct several regression analyses with the treatment as a dependent and each potential confounder as an independent variable. Furthermore, it will be investigated whether adding interaction terms or higher-order terms for continuous variables will improve the balance of the model. The Hausman test will be used to check the Independence of Irrelevant Alternatives (IIA) Assumption, which is the main assumption of multinomial regression analysis.[30] If this assumption is not met, multinomial probit analysis will be used instead.

The estimation of propensity weights will be conducted by the method proposed by McCaffrey.[31,32] The appropriateness of IPW analysis will be assessed by checking the overlap (positivity assumption). According to the positivity assumption, each patient should have a non-zero probability of being indicated to each treatment category. Lack of overlap in the distribution of observed pre-treatment characteristics between groups receiving different treatment indicates the positivity assumption is violated. This overlap will be checked visually. If poor overlap is identified, we will use Rubin's trimming method,[33] allowing discarding not-overlapping cases.

Further, pairwise balance in the distribution of all included variables between the three treatment groups will be tested with linear regression for continuous variables, logistic regression for binary, and multinomial logistic regression for nominal variables. The analysis will be done without and with adjustments. The propensity weights will be considered balanced if there are no statistically significant (p<0.05) differences between the likelihood of receiving a different type of treatment. If imbalances remain after weighting, a doubly robust estimation approach will be applied,[32] i.e. the imbalanced variables will be added to the model.

Whilst IPW will adjust for confounding at baseline, other time-varying adjustment will be applied to correct for the events between the baseline and the outcome. The proposed covariates are treatment with levothyroxine for developed hypothyroidism, log TSH levels and informative observations of number of GP visits in a unit of time.

Incidence rate ratios of outcomes will be calculated with Poisson regression and modelled adjusting for time-varying covariates as listed below. Time-to-event analysis with adjustments will be modelled. The appropriate model will be applied depending on the proportional hazard assumption's results: if the assumption holds – Cox proportional hazard regression will be used; if it is violated, alternative techniques such as time-varying measures in extended Cox models will be applied.

Proposed time-varying adjustments for both analyses will include post-index date data, i.e. treatment with levothyroxine for developed hypothyroidism, logTSH levels, and informative observations of number of GP visits in a unit of time. To allow for the fact that patients may die, a competing risks approach will be adopted for estimating comparative incidences for non-fatal events to allow for the impact of mortality as a competing risk.[22]

For peer review only - http://bmjopen.bmj.com/site/about/guidelines.xhtml

For peer review only

Sensitivity to missed radioiodine record

Whenever ATD patients develop permanent hypothyroidism, a radioiodine treatment will be assumed, as explained in the Exposure section. All analyses will be repeated to check sensitivity to this assumption, excluding patients in the ATD group who developed hypothyroidism following medical treatment being the only antithyroid treatment on record.

PATIENT AND PUBLIC INVOLVEMENT

Our patients with hyperthyroidism have been pivotal in formulating the research questions and informing the study design. The study idea originated from listening to patients and their concerns. As part of the consultation process, we held a meeting, advertised locally and nationally via the British Thyroid Foundation (BTF), with patients who had either previously been treated or were currently being treated for hyperthyroidism. During an open discussion, key themes were identified as important to patients across the treatment course of hyperthyroidism. At diagnosis, patients often reported that there was insufficient information to determine which treatment choice would be best for them, particularly regarding long-term treatment consequences; hence, a major driver of the study design is the examination of long term cardio-metabolic outcomes. The comment that “radioactive iodine will make me fat” was repeatedly mentioned in the group discussion. We were already interested in the potential issue of weight gain beyond premorbid weight, and this is something we will be investigating in this project. The main themes reported were that in addition to insufficient information to make a clear, informed treatment choice, patients did not feel that they were suitably counselled about weight regain nor that there was a mechanism in place to predict or modulate this potential effect. The insufficiency of information on the treatment choices and their long-term consequences was further confirmed in a BTF survey involving 353 patients. Among responders who received definitive treatment (n=167) who therefore had knowledge of post-definitive treatment procedure effects, a third would have not decided to proceed with their choice of treatment.

To continue guidance by patients, we have formed a patient group, which was engaged at the design stage and will continue to advise us during the project. Additionally, a BTF representative (JP) joined our team as a co-applicant to represent patients’ voices regularly throughout the research process. As the study progresses, the patient group and the patients’ representative will be involved in rationalising the design if any challenges are encountered, in discussing the implications and relevance of the emerging results and in finalising the key messages of the study to facilitate patient-centred dissemination. We will involve our patient partners in the dissemination of the results and the development of future avenues of research.

Additional guidance comes from the GP advisory group (SJF, PS), who have shared their experience and expertise from the front-end of data collection. This was especially important while establishing the feasibility of modelling and interpreting the weight changes based on the already recorded weight entries. The GP advice will be further utilised in the interpretation of results, dissemination process and the planning of further initiatives.

ETHICS AND DISSEMINATION

For peer review only - http://bmjopen.bmj.com/site/about/guidelines.xhtml

The study protocol has been approved by the Independent Scientific Advisory Committee (ISAC) in March 2021 (#20_000185). ISAC is a non-statutory expert advisory body established in 2006 by the Secretary of State for Health to provide scientific advice on research requests to access data provided by CPRD.

The primary aim is to inform clinical practice within the NHS and provide evidence for the future research in managing long-term consequences of hyperthyroidism and its treatment. The dissemination plan targets audiences at various levels: clinical and academic as well as patients and public. To address the needs of the former, we are planning to share the findings at the scientific endocrinology meetings and to publish them in leading open-access peer-reviewed academic journals. The British Thyroid Foundation (BTF), a national community of patients with thyroid conditions in the UK, will play a key role in disseminating the results of the study to the public, patients and their families and carers. The BTF will share results via traditional and social media channels.

CONTRIBUTION:

BT, JMH, KN, GNT, KRA and KB participated in the conception and the initial design of the study, with further substantial contributions from SJF, PS and JP. All authors participated in drafting the protocol and have approved the final version.

ACKNOWLEDGEMENT:

The authors would like to thank Prof. Daniel Lasserson for special contribution to the study protocol.

FUNDING:

This project is funded by the National Institute for Health Research (NIHR) under its Research for Patient Benefit (RfPB) Programme (Grant Number: NIHR200772). The views expressed are those of the authors and not necessarily those of the NIHR or the Department of Health and Social Care.

COMPETING INTERESTS:

BT, JMH, KN, GNT, JP, SJF, PS, KB have nothing to declare.

KRA has served as a paid consultant, providing unrelated methodological advice to: Abbvie, Amaris, Allergan, Astellas, AstraZeneca, Boehringer Ingelheim, Bristol-Meyers Squibb, Creativ-Ceutical, GSK, ICON/Oxford Outcomes, Ipsen, Janssen, Eli Lilly, Merck, NICE, Novartis, NovoNordisk, Pfizer, PRMA, Roche and Takeda, and has received research funding from Association of the British Pharmaceutical Industry (ABPI), European Federation of Pharmaceutical Industries & Associations (EFPIA), Pfizer and Sanofi. He is a Partner and Director of Visible Analytics Limited, a healthcare consultancy company.

For peer review only

For peer review only - http://bmjopen.bmj.com/site/about/guidelines.xhtml

REFERENCES

1. Vanderpump MP. The epidemiology of thyroid disease. Br Med Bull. 2011;99:39-51.
2. Boelaert K, Torlinska B, Holder RL, Franklyn JA. Older subjects with hyperthyroidism present with a paucity of symptoms and signs: a large cross-sectional study. J Clin Endocrinol Metab. 2010;95(6):2715-26.
3. Brandt F, Green A, Hegedus L, Brix TH. A critical review and meta-analysis of the association between overt hyperthyroidism and mortality. Eur J Endocrinol. 2011;165(4):491-7.
4. Abraham P, Avenell A, McGeoch SC, Clark LF, Bevan JS. Antithyroid drug regimen for treating Graves' hyperthyroidism. Cochrane Database Syst Rev. 2010(1):CD003420.
5. Ross DS, Burch HB, Cooper DS, Greenlee MC, Laurberg P, Maia AL, et al. 2016 American Thyroid Association Guidelines for Diagnosis and Management of Hyperthyroidism and Other Causes of Thyrotoxicosis. Thyroid. 2016;26(10):1343-421.
6. NICE. Hyperthyroidism.: National Institute for Health and Care Excellence; 2020 [13/04/2021]. Available from: https://cks.nice.org.uk/topics/hyperthyroidism/.
7. Boelaert K, Maisonneuve P, Torlinska B, Franklyn JA. Comparison of mortality in hyperthyroidism during periods of treatment with thionamides and after radioiodine. J Clin Endocrinol Metab. 2013;98(5):1869-82.
8. Ryodi E, Metso S, Huhtala H, Valimaki M, Auvinen A, Jaatinen P. Cardiovascular Morbidity and Mortality After Treatment of Hyperthyroidism with Either Radioactive Iodine or Thyroidectomy. Thyroid. 2018;28(9):1111-20.
9. Dale J, Daykin J, Holder R, Sheppard MC, Franklyn JA. Weight gain following treatment of hyperthyroidism. Clin Endocrinol (Oxf). 2001;55(2):233-9.
10. Rotondi M, Croce L, Pallavicini C, Manna LL, Accornero SM, Fonte R, et al. Body weight changes in a large cohort of patients subjected to thyroidectomy for a wide spectrum of thyroid diseases. Endocr Pract. 2014;20(11):1151-8.
11. Torlinska B, Nichols L, Mohammed MA, McCabe C, Boelaert K. Patients Treated for Hyperthyroidism Are at Increased Risk of Becoming Obese: Findings from a Large Prospective Secondary Care Cohort. Thyroid. 2019;29(10):1380-9.
12. Galindo RJ, Hurtado CR, Pasquel FJ, Garcia Tome R, Peng L, Umpierrez GE. National Trends in Incidence, Mortality, and Clinical Outcomes of Patients Hospitalized for Thyrotoxicosis With and Without Thyroid Storm in the United States, 2004-2013. Thyroid. 2019;29(1):36-43.
13. Metso S, Auvinen A, Salmi J, Huhtala H, Jaatinen P. Increased long-term cardiovascular morbidity among patients treated with radioactive iodine for hyperthyroidism. Clin Endocrinol (Oxf). 2008;68(3):450-7.
14. Okosieme OE, Taylor PN, Evans C, Thayer D, Chai A, Khan I, et al. Primary therapy of Graves' disease and cardiovascular morbidity and mortality: a linked-record cohort study. Lancet Diabetes Endocrinol. 2019;7(4):278-87.
15. Selmer C, Olesen JB, Hansen ML, Lindhardsen J, Olsen AMS, Madsen JC, et al. The spectrum of thyroid disease and risk of new onset atrial fibrillation: a large population cohort study. Bmj-British Medical Journal. 2012;345:12.
16. Baumgartner C, da Costa BR, Collet TH, Feller M, Floriani C, Bauer DC, et al. Thyroid Function Within the Normal Range, Subclinical Hypothyroidism, and the Risk of Atrial Fibrillation. Circulation. 2017;136(22):2100-16.
17. Gencer B, Collet TH, Virgini V, Bauer DC, Gussekloo J, Cappola AR, et al. Subclinical thyroid dysfunction and the risk of heart failure events: an individual participant data analysis from 6 prospective cohorts. Circulation. 2012;126(9):1040-9.
18. Thayakaran R, Adderley NJ, Sainsbury C, Torlinska B, Boelaert K, Sumilo D, et al. Thyroid replacement therapy, thyroid stimulating hormone concentrations, and long term health outcomes in patients with hypothyroidism: longitudinal study. BMJ. 2019;366:l4892.

For peer review only - http://bmjopen.bmj.com/site/about/guidelines.xhtml

19. Herrett E, Gallagher AM, Bhaskaran K, Forbes H, Mathur R, van Staa T, et al. Data Resource Profile: Clinical Practice Research Datalink (CPRD). Int J Epidemiol. 2015;44(3):827-36.
20. Marston L, Carpenter JR, Walters KR, Morris RW, Nazareth I, Petersen I. Issues in multiple imputation of missing data for large general practice clinical databases. Pharmacoepidemiol Drug Saf. 2010;19(6):618-26.
21. Royston P, White IR. Multiple Imputation by Chained Equations (MICE): Implementation in Stata. Journal of Statistical Software. 2011;45(4):1-20.
22. Crowther MJ, Abrams KR, Lambert PC. Joint Modeling of Longitudinal and Survival Data. The Stata Journal. 2013;13(1):165-84.
23. Erler NS, Rizopoulos D, Jaddoe VW, Franco OH, Lesaffre EM. Bayesian imputation of time-varying covariates in linear mixed models. Stat Methods Med Res. 2019;28(2):555-68.
24. Erler NS, Rizopoulos D, Lesaffre EM. JointAI: Joint Analysis and Imputation of Incomplete Data in R. arXiv [Internet]. 2020 20/06/2021. Available from: https://arxiv.org/abs/1907.10867.
25. Erler NS, Rizopoulos D, Rosmalen Jv, Jaddoe VWV, Franco OH, Lesaffre EMEH. Dealing with missing covariates in epidemiologic studies: a comparison between multiple imputation and a full Bayesian approach. Statistics in Medicine. 2016;35(17):2955-74.
26. World Health Organisation. Body mass index - BMI. 2021 [30/03/2021]. Available from: https://www.euro.who.int/en/health-topics/disease-prevention/nutrition/a-healthy-lifestyle/body-mass-index-bmi.
27. Yu Y, Zhang M, Shi X, Caram MEV, Little RJA, Mukherjee B. A comparison of parametric propensity score-based methods for causal inference with multiple treatments and a binary outcome. Statistics in Medicine. 2021;40(7):1653-77.
28. Lopez MJ, Gutman R. Estimation of Causal Effects with Multiple Treatments: A Review and New Ideas. Statistical Science. 2017;32(3):432-54.
29. Imbens G. The role of the propensity score in estimating dose-response functions. Biometrika. 2000;87(3):706-10.
30. Spreeuwenberg MD, Bartak A, Croon MA, Hagenaars JA, Busschbach JJV, Andrea H, et al. The Multiple Propensity Score as Control for Bias in the Comparison of More Than Two Treatment Arms: An Introduction From a Case Study in Mental Health. Medical Care. 2010;48(2):166-74.
31. Cefalu M, Buenaventura M. Propensity Scores for Multiple Treatments: A Tutorial on the MNPS Command for Stata Users: RAND Corporation; 2017.
32. McCaffrey DF, Griffin BA, Almirall D, Slaughter ME, Ramchand R, Burgette LF. A tutorial on propensity score estimation for multiple treatments using generalized boosted models. Stat Med. 2013;32(19):3388-414.
33. Rubin DB. Estimating causal effects of treatments in randomized and nonrandomized studies. Journal of Educational Psychology. 1974;66(5):688-701.

Figure 1: Directed acyclic graph illustrating confounding in the study. The wide dark arrow indicates the relationship of interest. ATD: antithyroid drugs, TFT: thyroid function test, IMD: Index of Multiple Deprivation

For peer review only - http://bmjopen.bmj.com/site/about/guidelines.xhtml

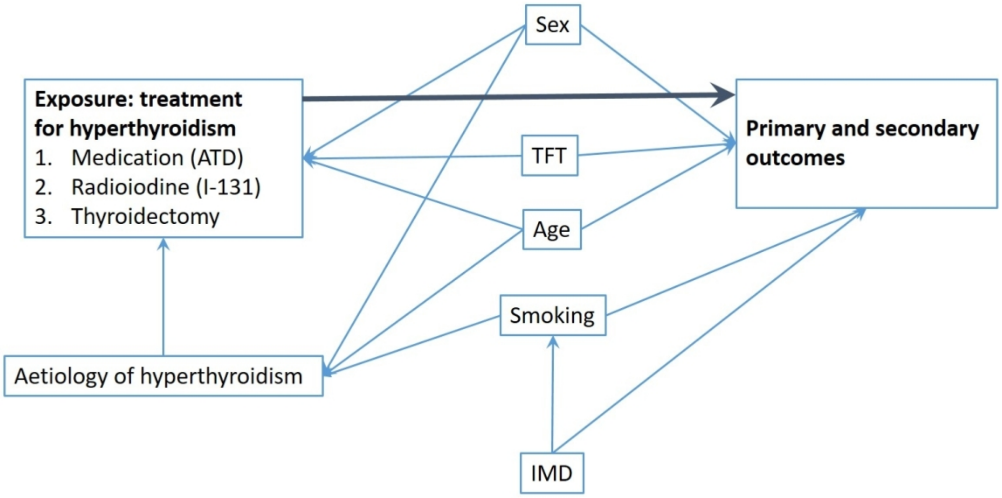

Figure 1: Directed acyclic graph (DAG) illustrating confounding in the study. The wide dark arrow indicates the relationship of interest. ATD: antithyroid drugs, TFT: thyroid function test, IMD: Index of Multiple Deprivation

259x130mm (150 x 150 DPI)

APPENDIX: Medical codes used for data search

Table 1: Read codes for diagnosis of hyperthyroidism

Read code	Description
C02..00	Thyrotoxicosis
C02..11	Hyperthyroidism
C02..12	Toxic goitre
C020.00	Toxic diffuse goitre
C020.11	Basedow's disease
C020.12	Graves' disease
C020000	Toxic diffuse goitre with no crisis
C020100	Toxic diffuse goitre with crisis
C020200	Thyroid-associated dermopathy
C020z00	Toxic diffuse goitre NOS
C021.00	Toxic uninodular goitre
C021000	Toxic uninodular goitre with no crisis
C021100	Toxic uninodular goitre with crisis
C021z00	Toxic uninodular goitre NOS
C022.00	Toxic multinodular goitre
C022000	Toxic multinodular goitre with no crisis
C022100	Toxic multinodular goitre with crisis
C022z00	Toxic multinodular goitre NOS
C023.00	Toxic nodular goitre unspecified
C023000	Toxic nodular goitre unspecified with no crisis
C023100	Toxic nodular goitre unspecified with crisis
C023z00	Toxic nodular goitre NOS
C024.00	Thyrotoxicosis from ectopic thyroid nodule
C024000	Thyrotoxicosis from ectopic thyroid nodule with no crisis
C024100	Thyrotoxicosis from ectopic thyroid nodule with crisis
C024z00	Thyrotoxicosis from ectopic thyroid nodule NOS
C02y.00	Thyrotoxicosis of other specified origin
C02y.11	Factitia thyrotoxicosis
C02y000	Thyrotoxicosis of other specified origin with no crisis
C02y100	Thyrotoxicosis of other specified origin with crisis
C02y200	Thyrotoxicosis factitia
C02y300	Thyroid crisis
C02yz00	Thyrotoxicosis of other specified origin NOS
C02z.00	Thyrotoxicosis without mention of goitre or other cause
C02z000	Thyrotoxicosis without mention of goitre or cause no crisis
C02z100	Thyrotoxicosis without mention of goitre, cause with crisis
C02zz00	Thyrotoxicosis NOS

For peer review only

Table 2: Codes for the treatment group allocation

Treatment group allocation	Coding system		
	Read	BNF	OPCS-4
**Medication:**			
Carbimazole		06.02.02.00	
Propylthiouracil		06.02.02.00	
**Radioiodine:**			
Radioactive drug therapy	5A16.00		X65.5
I131 radiotherapy	5A16.11		
Iodine 131 radiotherapy	5A16.12		
**Thyroidectomy:**			
Thyroid gland operations	711..12		B12.8
Thyroidectomy operations	7110.00		
Excision of thyroid gland operations	7110.11		
Total thyroidectomy	7110000		B08.1
Subtotal thyroidectomy	7110100		B08.2
Bilateral subtotal thyroidectomy	7110111		B08.2
Hemithyroidectomy	7110200		B08.3
Lobectomy of thyroid gland NEC	7110300		B08.4
Isthmectomy of thyroid gland	7110400		B08.5
Partial thyroidectomy NEC	7110500		B08.6
Thyroidectomy NEC	7110600		B08.6
Other specified thyroidectomy	7110y00		B08.8
Thyroidectomy NOS	7110z00		B08.9

For peer review only

Table 3: Outcomes codes

Outcome	Coding system	
	**ICD-10**	**Read codes**
obesity		22K5.00, 66C..00, 66CZ.00, C38..00, C380.00, C380.00, C380000, C380100, C380200, C380300, C380400, C380500, C38y011, C38z.00, C38z000
nonfatal myocardial infarction (composite of MACE)	I21-I22	n/a
nonfatal stroke (composite of MACE)	I64	n/a
congestive heart failure	I50	n/a
ischaemic heart disease	I20-I25	n/a
stroke and transient ischaemic attack (TIA)	I60-I69	n/a
type 2 diabetes mellitus	E11	C109*

MACE: major adverse cardiovascular event; TIA: transient ischemic attack

For peer review only
