# Supplementary material for: wEight chanGes, caRdio-mEtabolic risks and morTality in patients with hyperthyroidism (EGRET): a protocol for a CPRD–HES linked cohort study
Source: BMJ Open. 2021 Oct 1;11(10):e055219. doi: 10.1136/bmjopen-2021-055219 (PMC8488707; doi:10.1136/bmjopen-2021-055219)
Supplement: Reviewer comments [file bmjopen-2021-055219.reviewer_comments.pdf]

PEER REVIEW HISTORY

BMJ Open publishes all reviews undertaken for accepted manuscripts. Reviewers are asked to complete a checklist review form (**http://bmjopen.bmj.com/site/about/resources/checklist.pdf**) and are provided with free text boxes to elaborate on their assessment. These free text comments are reproduced below.

ARTICLE DETAILS

**TITLE (PROVISIONAL)**	wEight chanGes, caRdio-mEtabolic risks and morTality in patients with hyperthyroidism (EGRET): a protocol for a CPRD–HES linked cohort study
**AUTHORS**	Torlinska, Barbara; Hazlehurst, Jonathan; Nirantharakumar, Krishnarajah; Thomas, Neil; Priestley, Julia; Finnikin, Samuel; Saunders, Philip; Abrams, Keith; Boelaert, K

This article was not externally reviewed at BMJ Open. Protocols that have been independently assessed prior to submission to BMJ Open are usually fast-tracked to publication on the grounds that further substantial changes will not be possible. This independent assessment will usually be external, independent review for both a substantial grant award from a non-commercial or government funder and ethics approval.
